# Transcription factor EB overexpression prevents neurodegeneration in experimental synucleinopathies

**DOI:** 10.1172/jci.insight.129719

**Published:** 2019-08-22

**Authors:** Marie-Laure Arotcarena, Mathieu Bourdenx, Nathalie Dutheil, Marie-Laure Thiolat, Evelyne Doudnikoff, Sandra Dovero, Andrea Ballabio, Pierre-Olivier Fernagut, Wassilios G. Meissner, Erwan Bezard, Benjamin Dehay

**Affiliations:** 1 Université de Bordeaux, Institut des Maladies Neurodégénératives, UMR 5293, F-33000 Bordeaux, France.; 2 CNRS, Institut des Maladies Neurodégénératives, UMR 5293, F-33000 Bordeaux, France.; 3 Telethon Institute of Genetics and Medicine (TIGEM), Pozzuoli (Naples), Italy.; 4 Department of Translational Medicine, Federico II University, Naples, Italy.; 5 Department of Molecular and Human Genetics, Ian and Dan Duncan Neurological Research Institute, Baylor College of Medicine, Houston, Texas, USA.; 6 Laboratoire de Neurosciences Expérimentales et Cliniques, INSERM U-1084, Université de Poitiers, Poitiers, France.; 7 Service de Neurologie, Centre Hospitalier Universitaire de Bordeaux, Bordeaux, France.

**Keywords:** Neuroscience, Therapeutics, Autophagy, Gene therapy, Parkinson’s disease

## Abstract

The synucleinopathies Parkinson’s disease (PD) and Multiple system atrophy (MSA) — characterized by α-synuclein intracytoplasmic inclusions into, respectively, neurons and oligodendrocytes — are associated with impairment of the autophagy-lysosomal pathways (ALP). Increased expression of the master regulator of ALP, transcription factor EB (TFEB), is hypothesized to promote the clearance of WT α-synuclein and survival of dopaminergic neurons. Here, we explore the efficacy of targeted TFEB overexpression either in neurons or oligodendrocytes to reduce the pathological burden of α-synuclein in a PD rat model and a MSA mouse model. While TFEB neuronal expression was sufficient to prevent neurodegeneration in the PD model, we show that only TFEB oligodendroglial overexpression leads to neuroprotective effects in the MSA model. These beneficial effects were associated with a decreased accumulation of α-synuclein into oligodendrocytes through recovery of the ALP machinery. Our study demonstrates that the cell type where α-synuclein aggregates dictates the target of TFEB overexpression in order to be protective, paving the way for adapted therapies.

## Abstract 2

Targeted overexpression of transcription factor EB (TFEB) decreases accumulation of α-synuclein and prevents neurodegeneration in animal models of synucleinopathies.

## Graphical abstract

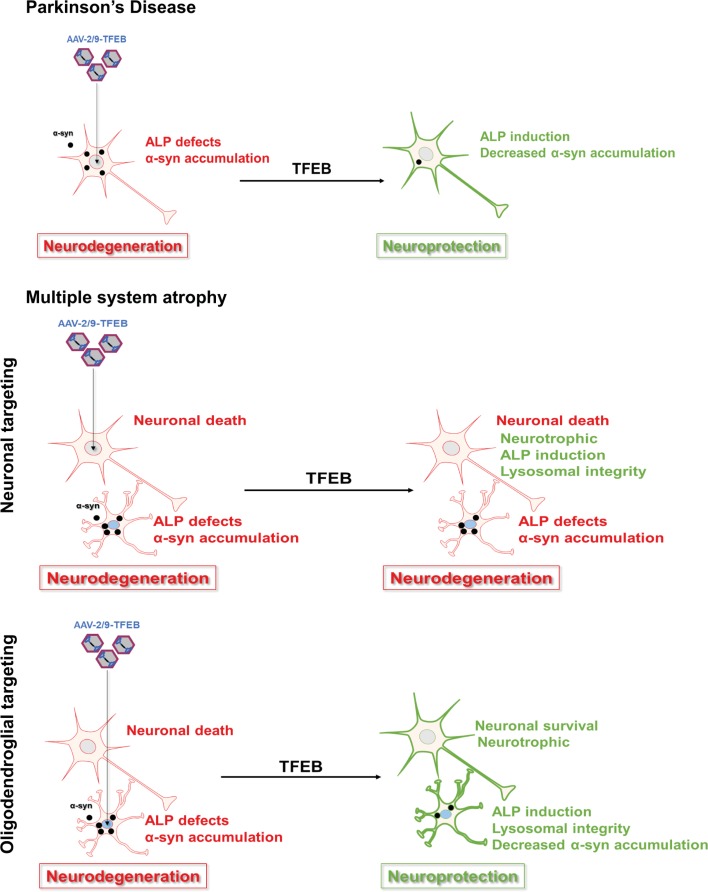

## Introduction

Synucleinopathies are a heterogenous group of neurodegenerative diseases characterized by the formation of α-synuclein (α-syn) aggregates. Parkinson’s disease (PD) is the second most common neurodegenerative disorder after Alzheimer’s disease and is characterized by motor and nonmotor symptoms. The motor symptomatology is mainly associated with profound dopamine depletion in the striatum due to the loss of mesencephalic dopaminergic neurons. The main neuropathological hallmark of PD is the presence of neuronal α-syn–positive intracytoplasmic inclusions named Lewy bodies (LB) in cell bodies and Lewy neurites in cell processes (1). Multiple system atrophy (MSA) is a rare, fast-progressing disease divided in 2 clinical phenotypes: (a) the MSA parkinsonian showing L-3,4-dihydroxyphenylalanine nonresponsive parkinsonian syndrome with bradykinesia, rigidity, and rest tremor due to a dopaminergic loss in the nigrostriatal pathway; and (b) the MSA cerebellar exhibiting cerebellar syndrome with gait, speech, and limb ataxia, and cerebellar oculomotor dysfunction caused by a neuronal loss in the olivopontocerebellar pathway. The neuropathological hallmark of MSA is the presence of α-syn–positive cytoplasmic inclusions, in oligodendrocytes, named glial cytoplasmic inclusions (2).

The presence of α-syn–positive aggregates suggests that a defect in α-syn degradation could play a role in the accumulation of the aggregated and misfolded proteins in these neurodegenerative disorders (3, 4). α-Syn degradation is ensured by the 2 protein degradation pathways: the ubiquitin-proteasome system (5) and the autophagy-lysosomal pathways (ALP) through both macroautophagy and chaperone-mediated autophagy (6–9). The ALP is a versatile cellular proteolytic system allowing the degradation of long-lived protein, protein aggregates, and abnormal organelles — among other cellular waste — through different mechanisms (10). Macroautophagy allows degradation after the formation and fusion of cargo-vesicles called autophagosomes carrying the material with lysosomes containing enzymatic material (10). Chaperone-mediated autophagy is a selective pathway allowing the degradation of protein after recognition of pentapeptide (KFERQ-like motif) by the cytosolic chaperone heat-shock cognate 70 kDa protein and delivery to the lysosome. Genetic and neuropathological evidence suggest that ALP defects are involved in the pathogenesis of neurodegenerative disorders. Relevant to synucleinopathies, several lines of evidence suggest that the assembly state of α-syn can affect the degradation machinery. In particular, monomeric and small soluble oligomeric forms of α-syn are degraded through the ubiquitin-proteasome system or chaperone-mediated autophagy, whereas larger oligomeric or aggregated forms are cleared by macroautophagy (11, 12).

Similar to PD, impairment of the ALP occurs in MSA. Postmortem studies showed that the microtubule-associated protein 1 light chain 3β (LC3B) autophagosome marker is increased and present within glial cytoplasmic inclusions in brains of MSA patients (13–15). Recently, it has also been demonstrated that the ALP is impaired in induced pluripotent stem cells–derived dopaminergic neurons from MSA patients, with decreased autophagic flux leading to further accumulation of nondegraded autophagosomes (16). Thus, defects in the ALP might be involved in the accumulation of α-syn in oligodendrocytes contributing to neurodegeneration in MSA. Enhancing ALP, thus, appears as a putative attractive approach for preventing accumulation of α-syn in synucleinopathies (10).

Transcription factor EB (TFEB) has emerged as a master activator of the autophagy machinery, as it regulates the expression of lysosomal genes through the Coordinated Lysosomal Expression and Regulation (CLEAR) signaling network, enhancing both lysosomal biogenesis and autophagy (17–22). In the context of PD, overexpression of TFEB has been shown to attenuate dopaminergic cell death and motor deficits induced by adeno-associated virus–mediated (AAV-mediated) overexpression of WT α-syn in rats through autophagy enhancement (23). Moreover, the important role of TFEB in the lysosomal-associated α-syn clearance has been elucidated in a genetic and chemical activation model of TFEB in vitro (24, 25). Recently, it has been also reported that overexpression of TFEB in 1-methyl-4-phenyl-1,2,3,6-tetrahydropyridine–intoxicated (MPTP-intoxicated) mice induces neurotrophic effects — in addition to its neuroprotective effects on dopaminergic neurons (26). Here, we investigate whether cell-specific induction of the ALP through AAV-mediated TFEB overexpression may exert beneficial effects in a viral-based rat model of PD and in the transgenic PLP α-syn mouse model of MSA by targeting cellular-specific overexpression of TFEB.

## Results

### TFEB overexpression induces neuroprotection in the A53T–α-Syn rat model of PD.

Since TFEB overexpression has been shown to be neuroprotective against AAV-mediated WT α-syn overexpression in rats (23), we first aimed at extending this observation through the assessment of the effects of increasing the ALP through TFEB overexpression in a rat model of PD based, this time, on the overexpression of human mutated A53T–α-syn. We used 2 AAV pseudotype 2/9 to deliver either human A53T–α-syn under the human synapsin-1 promoter with the cytomegalovirus (CMV) enhancer (CMVie/hSyn; AAV2/9-CMVie/hSyn-A53T–α-Syn) or 3×Flag-tagged murine TFEB (AAV2/9-CMV-mTFEB-3×Flag) under the CMV promoter. Both viruses were coinjected unilaterally in the substantia nigra pars compacta (SNpc). Four months after surgery, we validated that both viruses allowed expression of their respective transgenes in dopaminergic neurons, identified by tyrosine-hydroxylase (TH) immunostaining (Supplemental Figure 1, A–C; supplemental material available online with this article; https://doi.org/10.1172/jci.insight.129719DS1).

**Figure 1:**
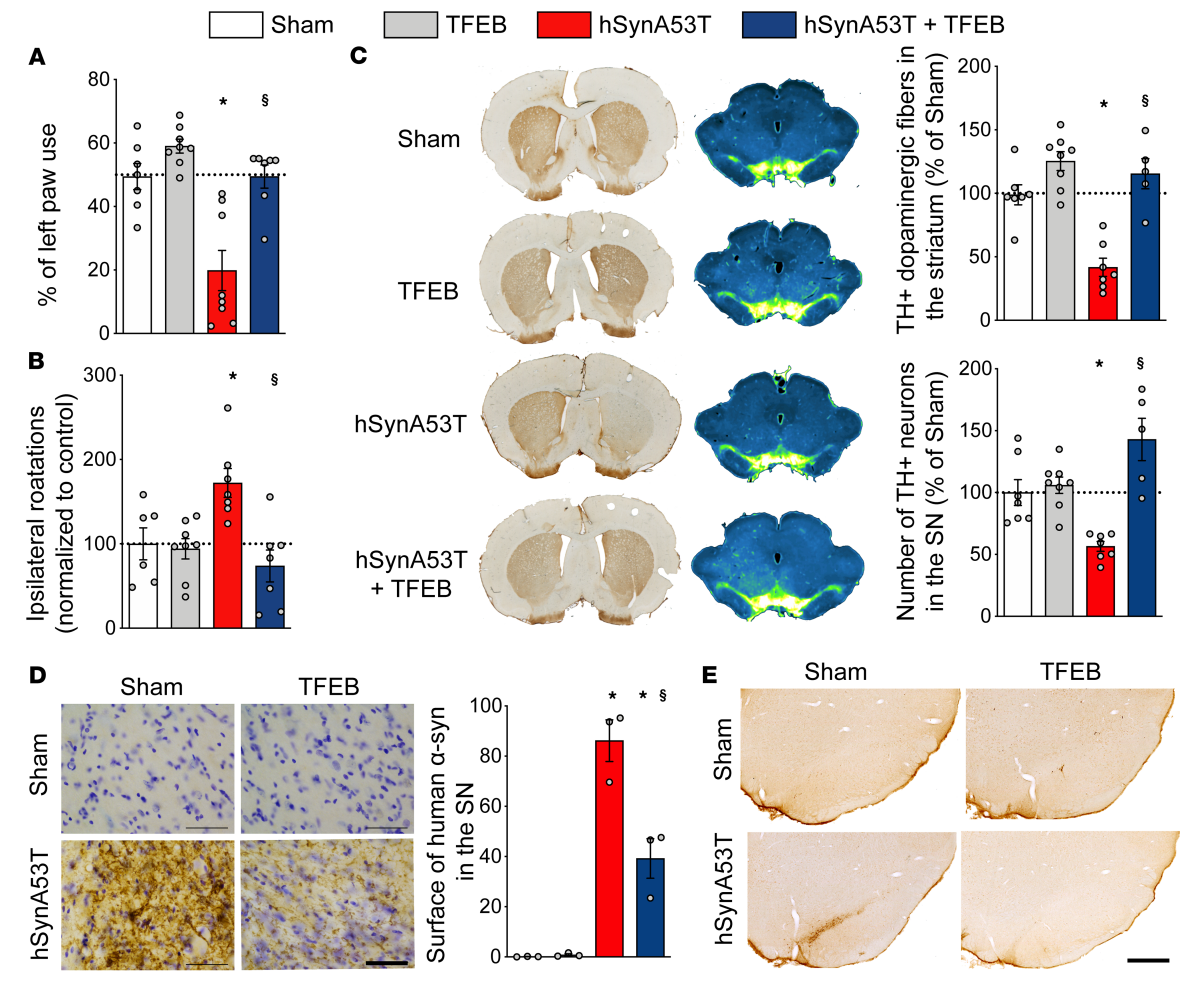
TFEB overexpression prevents mutant A53T–α-syn toxicity in a rat model of Parkinson’s disease. (**A**) TFEB overexpression restores the use of left paw in the cylinder test. (**B**) TFEB overexpression alleviates amphetamine-induced rotation behavior (1 mg/kg). (**C**) TFEB overexpression prevents α-syn–induced dopaminergic degeneration. Left lane and upper plot: representative images and quantification of striatal tyrosine hydroxylase (TH) staining. Right lane and lower plot: representative images of mesencephalic section of TH staining and stereological counting of TH-positive cells in the substantia nigra (SN). Inverted green fire blue lookup table was used to enhance visualization of the lesion. (**D**) Representative images and surface quantification of human α-syn staining in the SN. Scale bar: 50 μm. (**E**) Representative images of Serine129-phosphorylated α-syn in the SN. Scale bar: 500 μm. Data represent mean ± SEM. Comparisons were made using 1-way ANOVA and Bonferroni’s correction for multiple comparison, *n* = 7–8 per group. *P < 0.05 vs. sham-injected animals. ^$^*P* < 0.05 vs. hSynA53T-injected animals.

Four months after stereotactic surgery, we observed that coinjection of AAV-TFEB and AAV–A53T–α-syn was able to prevent behavioral impairments induced in this model of PD (Figure 1, A and B, and ref. 27). Indeed, TFEB overexpression maintained the use of the paw contralateral to the lesion in the cylinder test (F_[3,26]_ = 15.71, *P* < 0.0001) and significantly decreased amphetamine-induced rotation (F_[3,24]_ = 6.733, *P* = 0.0019). Immunohistological investigations revealed that the dopaminergic nigrostriatal tract was preserved when TFEB was coexpressed with mutated α-syn at both striatal dopaminergic terminals (Figure 1C; F_[3,23]_ = 21.07, *P* < 0.0001) and SNpc dopaminergic neurons (Figure 1C; F_[3,23]_ = 12.83, *P* < 0.0001) levels.

Further investigations showed that TFEB overexpression significantly decreased the pathological burden of α-syn with (a) a reduction of human mutated α-syn accumulation (Figure 1D; ~50% reduction; [F_(3,8)_ = 50.6, *P* < 0.0001]), (b) a dramatic decrease in pathological Serine129 phosphorylated α-syn (pS129–α-syn) staining levels in the SNpc (Figure 1E), and (c) a dampening of the induced astrogliosis in the SN (Supplemental Figure 1D). Altogether, these results both confirm and extend the proposal that overexpression of TFEB might be beneficial in experimental PD and also validate our TFEB transgene.

### Levels of TFEB are decreased in MSA patient brains.

**Figure 2:**
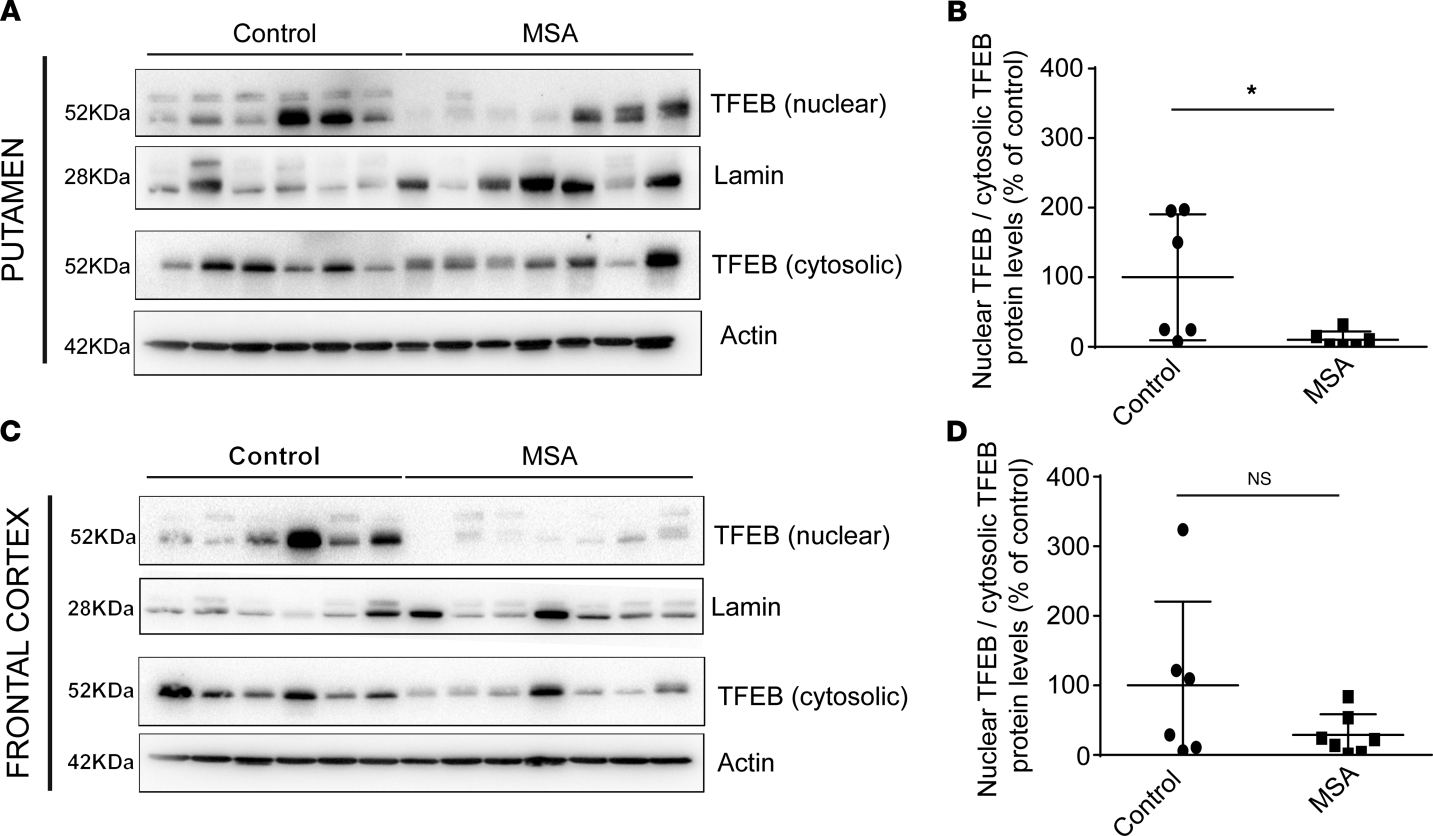
Reduced TFEB protein expression in MSA nuclear fractions in putamen and frontal cortex. (**A** and **B**) Representative immunoblot levels of TFEB in nuclear and cytosolic fractions from putamen of healthy individuals (*n* = 6) and MSA patients (*n* = 7). (**C** and **D**) TFEB immunoblot levels in nuclear and the cytosolic fractions from frontal cortex of age-matched healthy individuals and MSA patients. The term nuclear TFEB corresponds to the measured TFEB protein levels into the nuclear fraction normalized by Lamin protein levels. The term cytosolic TFEB corresponds to the measured TFEB protein levels into the cytosolic fraction normalized by Actin protein levels. The ratio of nuclear TFEB divided by cytosolic TFEB is then presented on the graph for each region. Data represent mean ± SEM. Comparisons were made using nonparametric *t* test. *P < 0.05 compared with healthy individuals.

Impaired TFEB function bas been previously reported in neurodegenerative diseases such as Alzheimer’s disease (28) and PD (23), but its potential impairment in MSA is still unknown. We thus determined whether defects in TFEB may also occur in MSA patients (Supplemental Table 1). Being a transcription factor, we investigated the subcellular localization of TFEB. We measured protein expression levels of TFEB in nuclear and cytosolic fractions from frontal cortex and putamen lysates of healthy individuals and MSA patients. We observed that TFEB protein levels were significantly reduced in the nuclear fractions in the putamen of MSA patients compared with healthy individuals (Figure 2, A and B, P** = 0.037) and, to a lesser extent, in the frontal cortex (Figure 2, C and D), a brain region less affected in MSA parkinsonian cases. These observations suggest a defect in TFEB nuclear translocation associated with a possible reduction in TFEB transcriptional activity, similar to other neurodegenerative diseases.

### Sustainable cell-specific expression of exogenous TFEB in the SN of PLP mice.

**Figure 3:**
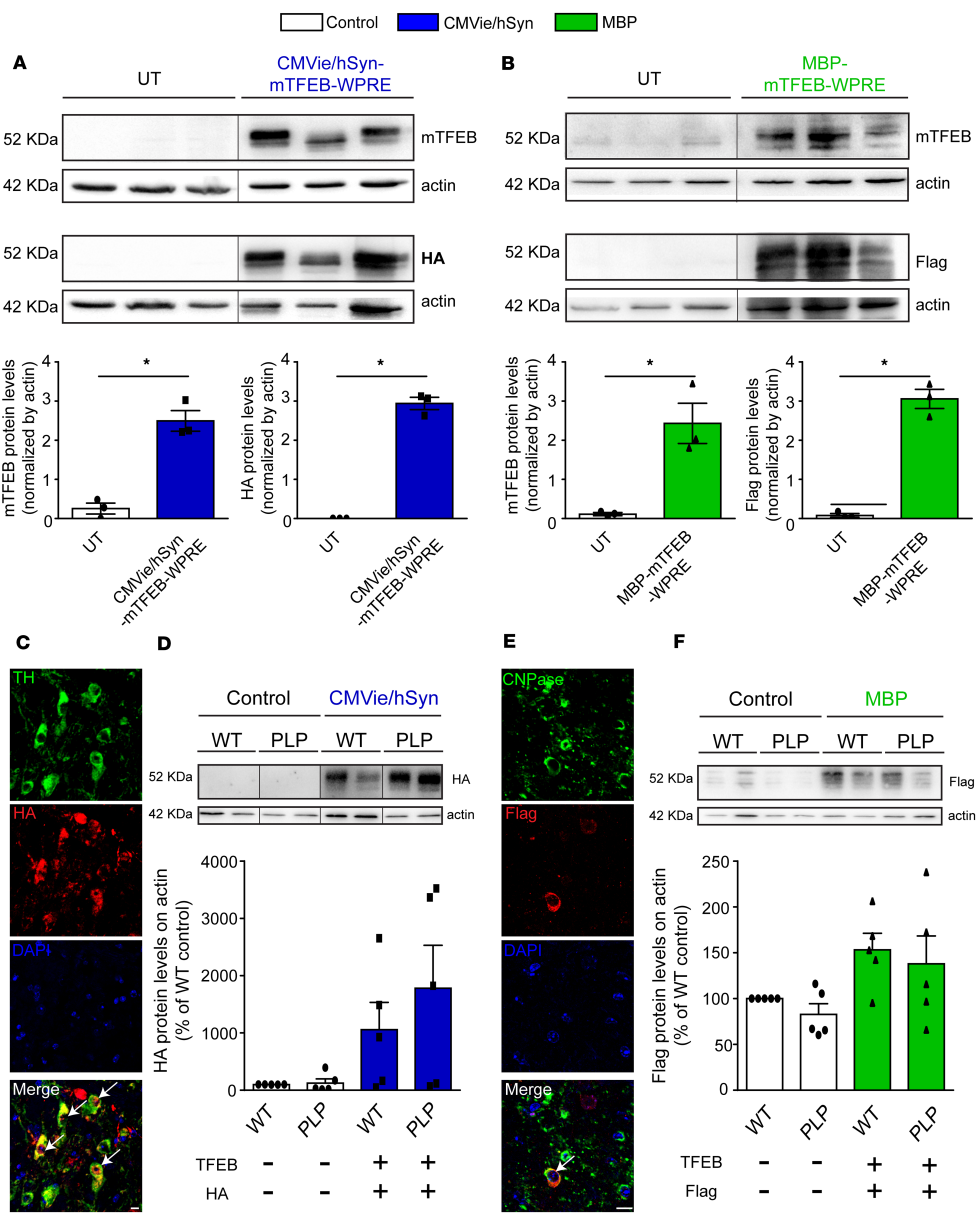
Sustainable TFEB transgene expression in vitro and in vivo. (**A**) Representative images (top) and quantification (bottom) of TFEB and HA tag immunoblotting in HEK293T cells transfected for 48 hours with the mTFEB-expressed plasmid under the neuronal CMVie/Synapsin promoter (CMVie/Synapsin-mTFEB-HA-WPRE). (**B**) Representative images (top) and quantification (bottom) of TFEB and Flag tag immunoblotting in HEK293T cells transfected for 48 hours with the mTFEB-expressed plasmid under the oligodendroglial promoter MBP (MBP-mTFEB-3×Flag-WPRE). Data represent mean ± SEM. Comparisons were made using nonparametric *t* test. *P < 0.05 compared with nontransfected cells. (**C**) Confocal images using HA tag and tyrosine hydroxylase (TH) antibodies in the ipsilateral SN of mice injected with the CMVie/Synpasin-mTFEB-HA virus 5 months after the injection. The white arrows indicate the presence of HA-tagged mTFEB signal into the nucleus of TH-positive neurons. Scale bar: 10 μm. (**D**) Representative images (top) and quantification (bottom) of HA tag immunoblotting in the ipsilateral SN of CMVie/Synapsin-mTFEB-HA–injected WT and PLP mice 5 months after the injection. *n* = 5 per group. Data represent mean ± SEM. Comparisons were made using 1-way ANOVA and Tukey’s correction for multiple comparisons. White bars, control; blue bars, CMVie/Synapsin-mTFEB-HA. (**E**) Confocal images using Flag tag and CNPase antibodies in the ipsilateral SN of mice injected with the MBP-mTFEB-3×Flag virus 5 months after the injection. The white arrow indicates the presence of Flag tag signals into the nucleus of CNPase-positive oligodendrocytes. Scale bar: 10 μm. (**F**) Representative images (top) and quantification (bottom) of Flag tag immunoblotting in the ipsilateral SN of MBP-mTFEB–injected WT and PLP mice 5 months after the injection. Data represent mean ± SEM. Comparisons were made using 1-way ANOVA and Tukey’s correction for multiple comparisons. White bars, control; green bars, MBP-mTFEB-3×Flag. In **A**, **B**, **D**, and **F**, lanes were run on the same gel but were noncontiguous.

To address the therapeutic potential of restoring TFEB levels in a clinically relevant mouse model of MSA, we aimed at overexpressing TFEB in a transgenic mouse model of MSA expressing human WT α-syn under the control of the oligodendrocyte-specific promoter PLP (hereafter referred as PLP mice) (29–33). In the PD-relevant experiments, human α-syn was expressed in SNpc neurons. In the MSA-relevant experiments, we compared the effects of neuronal vs. oligodendroglial TFEB overexpression. To this end, we employed the CMVie/hSyn promoter to drive neuron-specific expression of HA-tagged mTFEB (CMVie/hSyn-mTFEB-HA) and the myelin basic protein (MBP) promoter for oligodendrocyte-specific expression of 3×Flag-tagged mTFEB (MBP-mTFEB-3×Flag). To validate and determine the protein expression level of exogenous TFEB, we transiently expressed the 2 constructs in HEK293T cell lines and performed immunoblotting against TFEB, HA, and Flag (Figure 3, A and B). Transfected cells exhibited over 2-fold increase for each cell type–specific promoter with similar levels of expression between the 2 promoters (Figure 3, A and B).

AAV2/9-mediated transgene expression was then evaluated in vivo 5 months after intranigral injection in WT and PLP mice (Figure 3, C–F, and Supplemental Figure 2). Under the CMVie/hSyn promoter, TFEB expression was identified in the cytoplasm and the nucleus of nigral TH-positive dopaminergic cells (Figure 3C), confirming neuronal expression. After 5 months, TFEB protein levels were increased by 10-fold in comparison with control mice for CMVie/hSyn-mTFEB-HA–injected mice (Figure 3D). Likewise, viral vectors expressing TFEB under the MBP promoter showed transgene expression into the cytoplasm and the nucleus of nigral oligodendrocytes (Figure 3E) and 1.5-fold increase in MBP-mTFEB-3×Flag–injected mice (Figure 3F). We thus were able to drive the overexpression of TFEB in the SN of WT and PLP mice in a cell-specific manner. The nuclear localization of exogenous TFEB either in neurons or in oligodendrocytes demonstrated the long-term expression level in AAV-mTFEB–transduced cells and the efficiency of TFEB to be translocated to the nucleus to exert its transcriptional activity (34, 35).

### Oligodendroglial-targeted TFEB overexpression rescues dopaminergic neurodegeneration in PLP mice.

**Figure 4:**
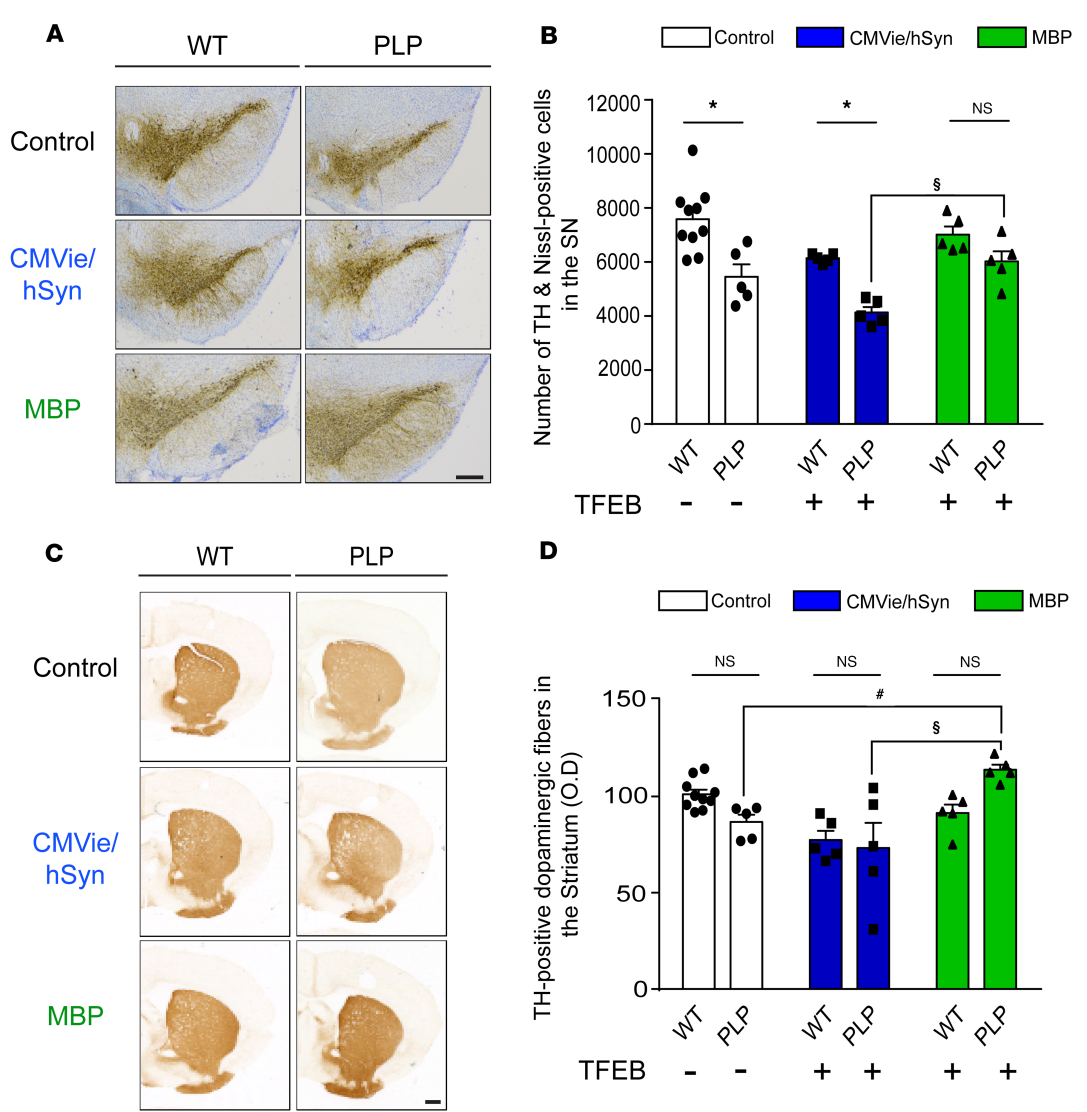
Specific oligodendroglial TFEB-targeted overexpression attenuates dopaminergic neurodegeneration in a MSA mouse model. (**A**) Representative images of TH staining in the SN of control, CMVie/hSyn-mTFEB–injected, and MBP-mTFEB–injected WT and PLP mice. Scale bar: 500 μm. (**B**) Number of TH- and Nissl-positive neurons counted by stereology in the SN of control, CMVie/hSyn-mTFEB–injected, and MBP-mTFEB–injected WT and PLP mice. (**C**) Representative images of TH staining into the striatum of control, CMVie/hSyn-mTFEB–injected, and MBP-mTFEB–injected WT and PLP mice. Scale bar: 500 μm. (**D**) Dot plot of mean gray-scale values of striatal TH immunoreactivity measured by optical density in the striatum of control, CMVie/hSyn-mTFEB–injected, and MBP-mTFEB–injected WT and PLP mice. *n* = 5 per group. White bars, control; blue bars, CMVie/hSyn-mTFEB-HA; green bars, MBP-mTFEB-3×Flag. Data represent mean ± SEM. Comparisons were made using 2-way ANOVA and Tukey’s correction for multiple comparisons. *P < 0.05 compared with WT mice. ^#^*P* < 0.05 compared with PLP control mice. ^$^*P* < 0.05 compared with CMVie/hSyn-mTFEB injected PLP mice.

Next, we hypothesized that cell type–specific expression of TFEB would differentially protect against neurodegeneration in PLP mice. As previously reported (29–33), noninjected control PLP mice displayed an approximately 30% loss of SN dopaminergic cells, as determined by stereological counting of SN TH- and Nissl-positive cells (Figure 4, A and B; F_[1,29]_ = 30.52, *P* = 0.0017) and a 15% loss of striatal dopaminergic terminals, as assessed by measurement of the optical density of TH-positive staining (Figure 4, C and D).

Interestingly, neuronal expression of TFEB in PLP mice did not afford neuroprotection, as we observed similar dopaminergic neurodegeneration both at the level of SN cell bodies and striatal terminals, compared with control mice (Figure 4, A–D). In contrast, oligodendroglial-targeted expression of TFEB attenuated neurodegeneration compared with neuronal-targeted TFEB in PLP mice, both at the level of SN dopaminergic neuron cell bodies and striatal dopaminergic neurons (Figure 4, A–D). Overall, these data indicated that only oligodendroglial-targeted TFEB overexpression can prevent nigrostriatal neurodegeneration in the PLP mouse model of MSA.

### Overexpression of TFEB in oligodendrocytes modestly prevents α-syn accumulation in the nigrostriatal pathway of PLP mice.

**Figure 5:**
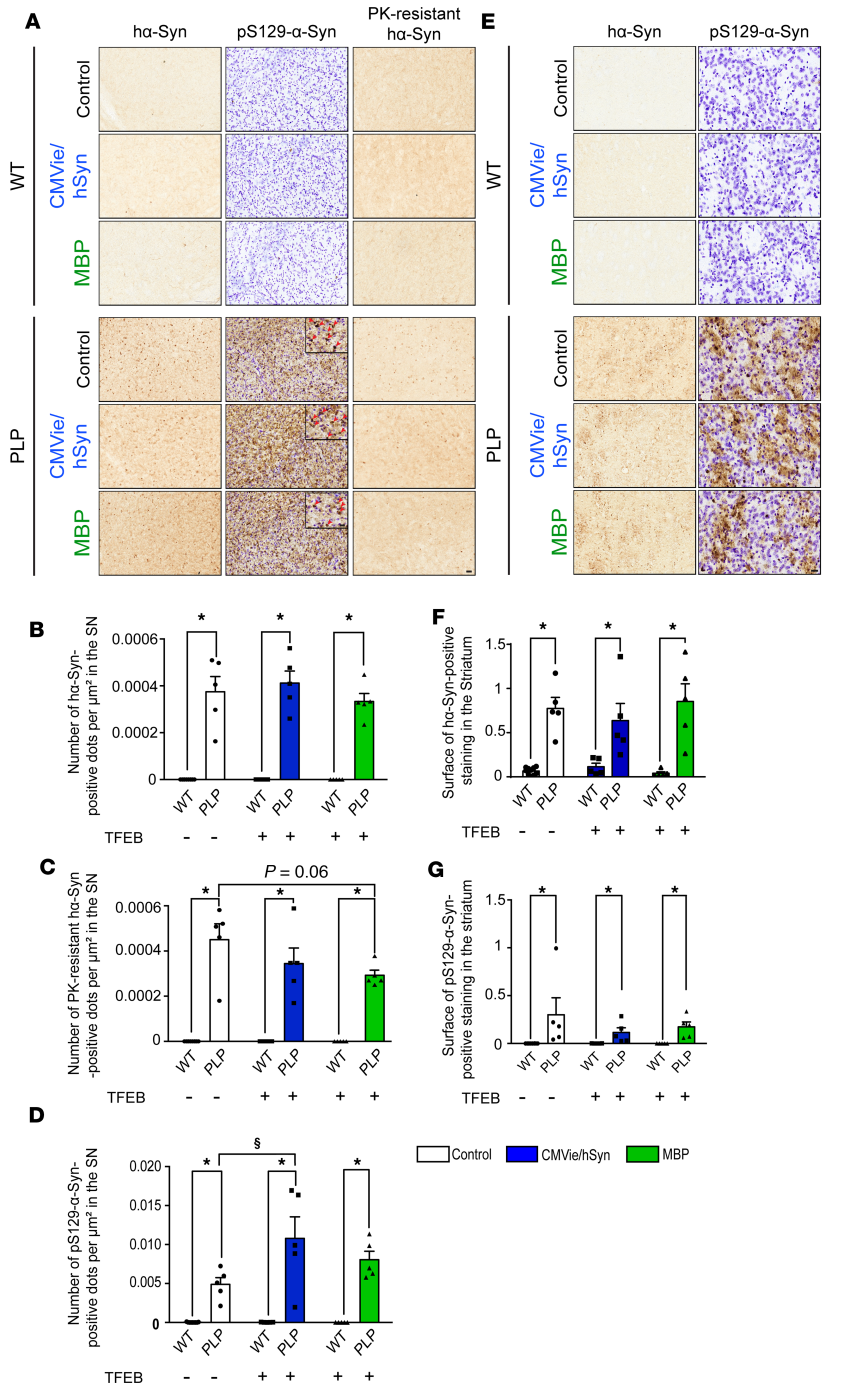
Oligodendroglial-targeted, but not neuronal TFEB–targeted, overexpression decreases the burden of aggregated forms of α-syn with no effect on phosphorylation at S129 in the nigrostriatal pathway of PLP mice. (**A**) Representative images of synucleinopathy in the SN of control, CMVie/hSyn-mTFEB–injected, and MBP-mTFEB–injected WT and PLP mice. Left panel: α-syn staining using the human-specific α-syn LB509 antibody. Middle panel: Serine129-phosphorylated α-syn staining using the EP1536Y antibody. Right panel: α-syn staining using human-specific α-syn LB509 antibody after proteinase K treatment. Scale bars: 50 μm; 20 μm (inset, middle panel). The red arrowheads show the S129-positive α-syn dots in the SN. (**B** and **C)** Number of α-synuclein–positive dots per μm² counted by stereology in the SN of control, CMVie/hSyn-mTFEB–injected, and MBP-mTFEB–injected WT and PLP mice without (**B**) and with (**C**) proteinase K treatment. (**D**) Number of Serine129-phosphorylated α-synuclein–positive dots per μm² in the SN of control, CMVie/hSyn-mTFEB–injected, and MBP-mTFEB–injected WT and PLP mice. (**E**) Representative images of synucleinopathy in the striatum of control, CMVie/hSyn-mTFEB–injected, and MBP-mTFEB–injected WT and PLP mice. Left panel: α-syn staining using the human-specific α-syn LB509 antibody. Right panel: Serine129-phosphorylated α-syn staining using the EP1536Y antibody. Scale bar: 100 μm. (**F**) Quantification of human α-syn LB509–positive immunostaining into the striatum of control, CMVie/hSyn-mTFEB–injected, and MBP-mTFEB–injected WT and PLP mice. (**G**) Quantification of Serine129-phosphorylated α-syn–positive immunostaining in the striatum of control, CMVie/hSyn-mTFEB–injected, and MBP-mTFEB–injected WT and PLP mice. *n* = 5 per group. White bars, control; blue bars, CMVie/hSyn-mTFEB-HA; green bars, MBP-mTFEB-3×Flag. Data represent mean ± SEM. Comparisons were made using 2-way ANOVA and Tukey’s correction for multiple comparisons. *P < 0.05 compared with WT animals. ^$^*P* < 0.05 compared with PLP control mice.

We next determined whether the neuroprotection associated with TFEB expression observed in PLP mice was also accompanied by a reduction of markers of synucleinopathy (i.e., our hypothesized primary target for enhanced ALP). We examined the effects of TFEB overexpression on α-syn species identified by a human-specific α-syn antibody in the absence or presence of proteinase K (PK) pretreatment (Figure 5A). PK treatment reveals the accumulation of misfolded PK-resistant α-syn aggregates, as reported in this transgenic PLP α-syn mouse model of MSA (29–33). Without PK treatment, no significant differences were observed regarding the number of α-syn–positive dots per μm² counted by stereology in the SN of injected PLP mice compared with control PLP animals (Figure 5B). Following PK digestion, however, AAV-MBP-mTFEB–injected PLP mice exhibited a marked reduction of PK-resistant aggregates in the SN compared with control mice (Figure 5C). However, no significant differences were observed in α-syn staining in the striatum in the 3 experimental groups (Figure 5, E and F).

To further characterize the effect of TFEB regarding α-syn pathology, we performed immunohistochemical investigations in the SN and the striatum with a phospho-specific Serine129 α-syn (pS129–α-syn) antibody used as a second indicator of pathological α-syn (Figure 5, A and E). We observed an increase in the number of pS129 α-syn dots per μm² in the SN of the CMVie/hSyn-mTFEB PLP mice compared with control PLP mice (Figure 5D). No significant differences were observed regarding pS129–α-syn immunostaining in the striatum (Figure 5G). Our results indicate that only oligodendroglial-targeted TFEB overexpression modestly reduces the burden of α-syn pathology, through the pathological PK-resistant species of α-syn, whereas neuronal-targeted TFEB had no effect or rather enhanced the accumulation of pS129–α-syn.

### Oligodendroglial TFEB–mediated neuroprotection is independent of astrogliosis and microgliosis in PLP mice.

**Figure 6:**
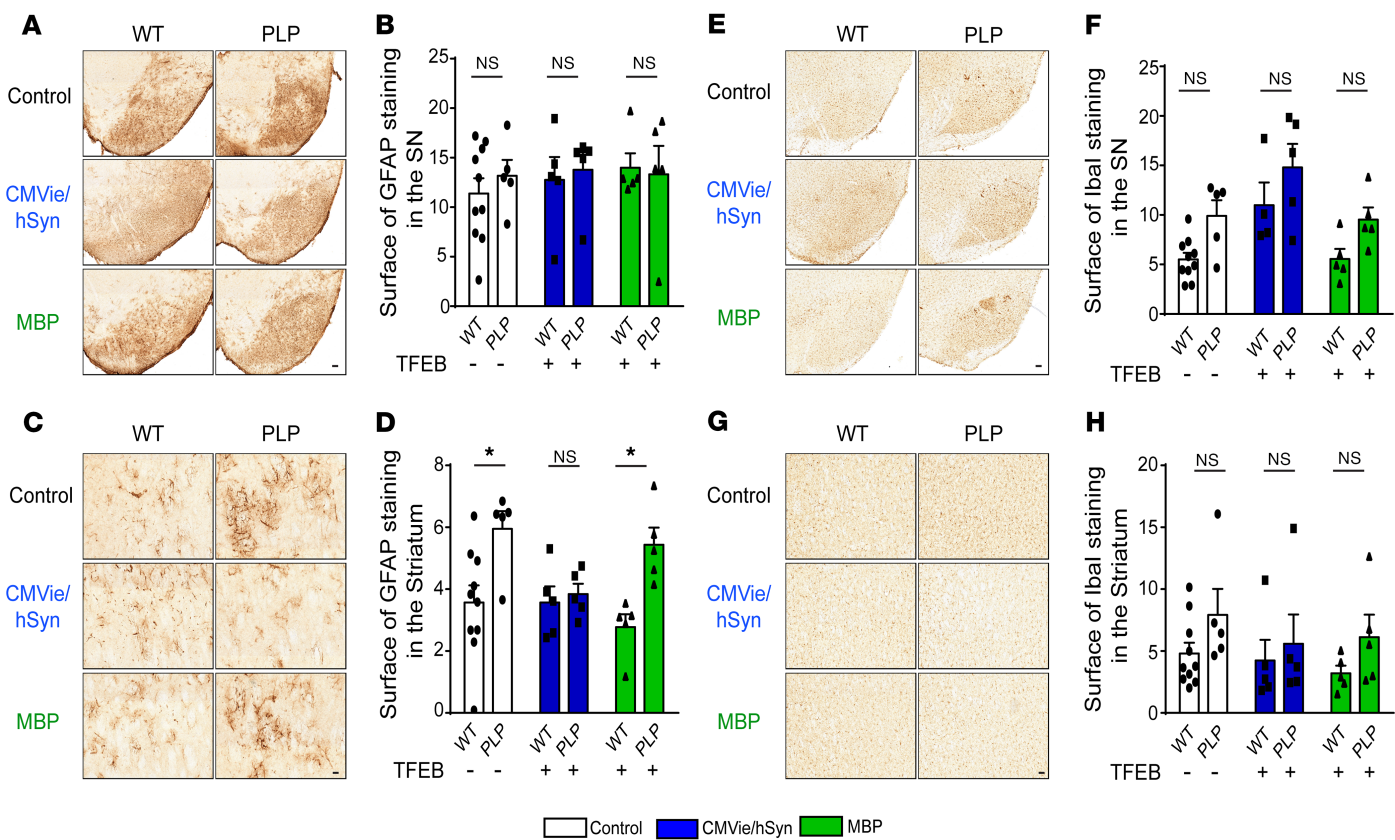
Lack of inflammatory reaction changes in the brain of TFEB-injected WT and PLP mice expressed under either the oligodendroglial or the neuronal promoter. (**A** and **B**) Representative images (**A**) and quantification (**B**) of GFAP-positive astrocytic immunostaining in the SN of control, CMVie/hSyn-mTFEB–injected, and MBP-mTFEB–injected WT and PLP mice. Scale bar: 200 μm. (**C** and **D**) Representative images (**C**) and quantification (**D**) of GFAP-positive astrocytic immunostaining in the striatum of control, CMVie/hSyn-mTFEB–injected, and MBP-mTFEB–injected WT and PLP mice. Scale bar: 50 μm. (**E** and **F**) Representative images (**E**) and quantification (**F**) of Iba1-positive microglial cells immunostaining in the SN of control, CMVie/hSyn-mTFEB–injected, and MBP-mTFEB- injected WT and PLP mice. Scale bar: 200 μm. (**G** and **H**) Representative images **(G)** and quantification **(H)** of Iba1-positive microglial cells immunostaining in the striatum of control, CMVie/hSyn-mTFEB–injected, and MBP-mTFEB–injected WT and PLP mice. Scale bar: 50 μm. *n* = 5 per group. White bars, control; blue bars, CMVie/hSyn-mTFEB-HA; green bars, MBP-mTFEB-3×Flag. Data represent mean ± SEM. Comparisons were made using 2-way ANOVA and Tukey’s correction for multiple comparisons. *P < 0.05 compared with WT animals.

Given the importance of the astroglial and microglial reaction in neurodegeneration, we next assessed the extent of astrogliosis and microgliosis using glial fibrillary acidic protein (GFAP) and ionized calcium-binding adapter molecule 1 (Iba1) immunostaining, respectively. No significant differences were observed between the 3 experimental groups regarding GFAP immunoreactivity in the SN (Figure 6, A and B). At the striatal level, we obtained a significant astrogliosis between PLP and WT mice, which was reduced in the CMVie/hSyn-mTFEB group, suggesting a decrease in astrogliosis following neuronal expression of TFEB (Figure 6, C and D), reminiscent of what we observed in the A53T–α-syn rat model of PD (Supplemental Figure 1D). Regarding microglia proliferation, no significant differences were observed in microglial Iba1 immunoreactivity in the SN (Figure 6, E and F) and in the striatum (Figure 6, G and H). These results suggest that targeted TFEB overexpression did not change the astrogliosis and microgliosis in the PLP mouse model of MSA.

### TFEB expression induces neurotrophic effects.

**Figure 7:**
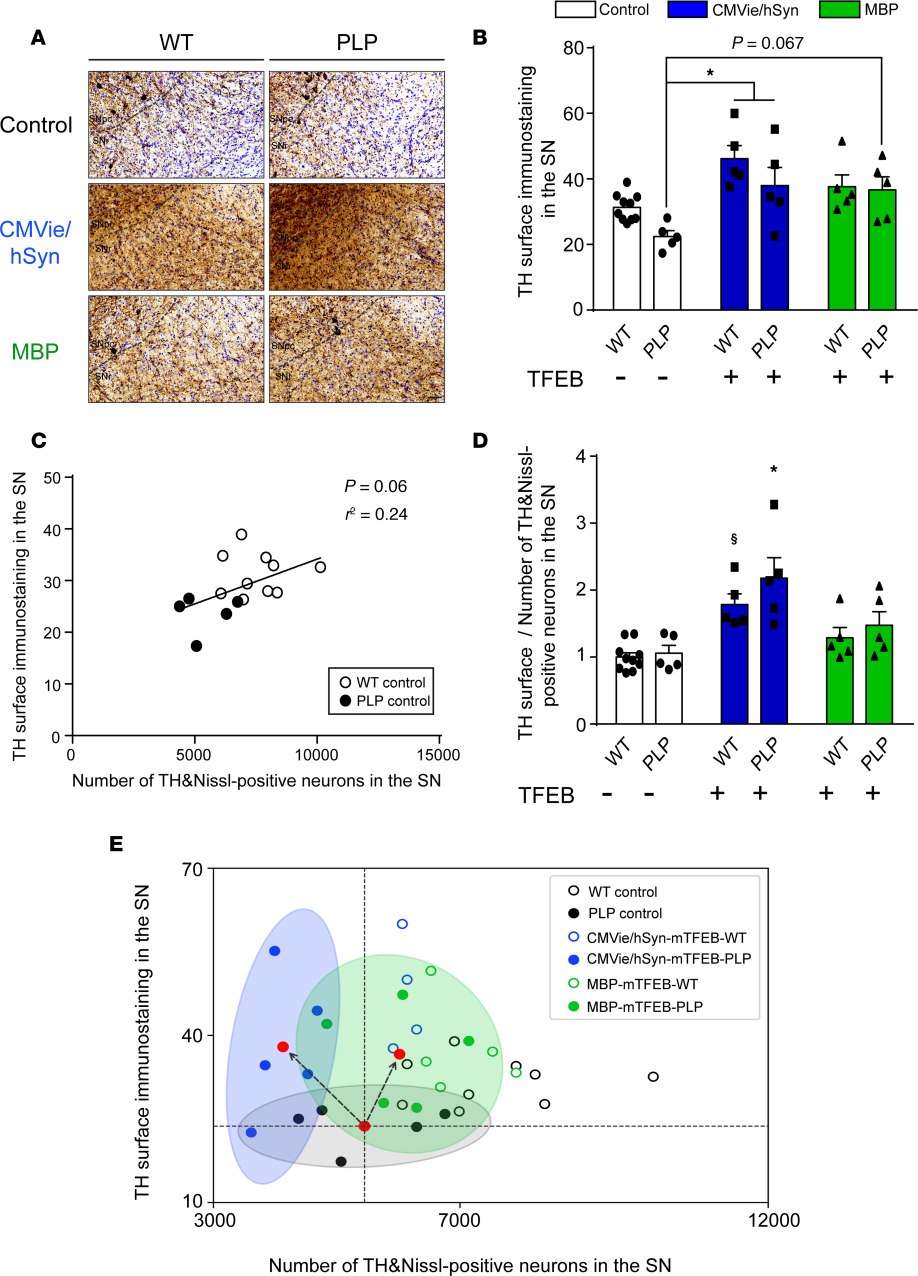
Both oligodendroglial– and neuronal TFEB–targeted overexpression induce neurotrophic effects in the brains of PLP mice. (**A** and **B**) Representative images (**A**) and quantification (**B**) of TH-positive immunostaining in the ipsilateral SN of control, CMVie/hSyn-mTFEB–injected, and MBP-mTFEB–injected WT and PLP mice. Scale bar: 50 μm. (**C**) Linear regression between TH-positive immunostaining and TH- and Nissl-positive cells in the SN of control WT (empty dark dots) and PLP mice (full dark dots). (**D**) Ratio of TH-positive immunostaining in the ipsilateral SN divided by the number of TH- and Nissl-positive neurons into the ipsilateral SN of control, CMVie/hSyn-mTFEB–injected, and MBP-mTFEB- injected WT and PLP mice. *n* = 5 per group. White bars, control; blue bars, CMVie/hSyn-mTFEB-HA; green bars, MBP-mTFEB-3×Flag. (**E**) Scatter plot of the value of TH surface immunostaining and the number of TH- and Nissl-positive neurons into the ipsilateral SN of control, CMVie/hSyn-mTFEB–injected, and MBP-mTFEB–injected WT and PLP mice. Each dot represents 1 animal. Red dot corresponds to the center of mass of each experimental group, and the ellipses represent the 95% CI around the center of mass: PLP control (black); CMVie/hSyn-mTFEB PLP (blue); MBP-mTFEB-PLP (green). Dashed lines are arbitrarily centered on the center of mass of PLP control group to distinguish between neurotrophic effect (toward upper left quadrant), neuroprotection (lower right quadrant), and a combination of both (upper right quadrant) in AAV-injected PLP groups; black arrows represent the direction of the change. Data represent mean ± SEM. Comparisons were made using 2-way ANOVA and Tukey’s correction for multiple comparisons. *P < 0.05 compared with control PLP animals. ^$^*P* < 0.05 compared with control WT animals.

Since TFEB expression was previously associated with neurotrophic effects (26), we wondered whether the neuroprotective effects observed upon oligodendroglial expression of TFEB may be explained by neurotrophic support. As a proxy for branching, we quantified TH-positive immunoreactive staining in the SN, reflecting the surface occupied by both the dopaminergic soma and the dopaminergic fibers. Overexpressing TFEB in either neurons or oligodendrocytes led to an increase of nigral TH immunoreactivity, suggesting a possible expansion of dopaminergic dendrites (Figure 7, A and B [F_(2,29)_ = 11.95; CMVie/hSyn-mTFEB PLP vs. control PLP, *P* = 0.0367; MBP-mTFEB PLP vs. control PLP, *P* = 0.0673]). We then confirmed this hypothesis in vitro by differentiating BE(2)-M17 human dopaminergic neuroblastoma cells with retinoic acid to induce a neuronal dendrite expansion phenotype (36) prior to transfection with the different TFEB-expressing plasmids. We observed that TFEB-transfected cells presented an increase in dendritic length measured by Simple Neural Tracer segmentation analysis, compared with nontransfected cells (Supplemental Figure 3, A and B; F_[2,239]_ = 41.6, *P* < 0.0001). These results confirmed that overexpression of TFEB in neuronal cells increases process length and confirms neurotrophic effect observed in vivo.

The PI3K/Akt pathway is a relevant signaling pathway for the mediation of neurotrophic activity (37). We thus measured the relative amount of Akt, as well as its Ser473-phosphorylated/activated form (P-Akt), by immunoblotting. The total amount of Akt protein was increased only when TFEB was overexpressed in oligodendrocytes (F_[2,24]_ = 8.47, *P* = 0.012), whereas no difference was observed for its phosphorylated form or for the ratio P-Akt/Akt (Supplemental Figure 3, C–F). These results indicated that oligodendroglial TFEB–mediated neurotrophic effects took place, at least in part, mediated through the regulation of Akt expression. No effect on the Akt signaling pathway was observed after neuronal-targeted TFEB expression, suggesting that the neurotrophic effect observed in this group was independent of the PI3K/Akt pathway activation.

Under basal WT and PLP mutant conditions, the number of TH- and Nissl-positive cells correlated with the surface of TH-positive immunostaining in the SN, suggesting that the PLP model showed dopaminergic neuronal soma and fiber loss in the SN (Figure 7C; F_[1,13]_ = 5.45, *P* = 0.06, r² = 0.24). Interestingly, the assessment of the relative contribution of TFEB-mediated neurotrophic (i.e., TH-positive immunostaining) vs. neuroprotective (i.e., number of TH- and Nissl-positive cells) in the SN revealed differential processes on the dopaminergic system according to the targeted cell type. Thus, overexpressing TFEB in oligodendrocytes led to both neuroprotective and neurotrophic effects (Figure 7, D and E; PLP control vs. MBP-mTFEB PLP mice), while neuronal overexpression of TFEB led only to neurotrophic effects, which were not sufficient to prevent neurodegeneration (Figure 7, D and E; PLP control vs. CMVie/hSyn-mTFEB PLP mice).

### Neuronal and oligodendroglial-targeted TFEB induces the autophagic clearance machinery.

Since TFEB is a master gene in the regulation of ALP (17–20, 22), we hypothesized that TFEB overexpression could lead to a recovery of the lysosomal machinery through lysosomal biogenesis and autophagy induction, which could enhance α-syn clearance. We first analyzed mRNA expression levels from SN patches showing that the *Tfeb* downstream target gene *Mcoln1* transcript expression is upregulated in MBP-mTFEB PLP–injected mice (Supplemental Figure 4A) associated with an upregulation of the autophagy-related *Tfeb* targets such as *Map1lc3a*, *Lamp2b*, *CtsB*, and *CtsF* genes (Supplemental Figure 4, B–E), suggesting a positive regulation of autophagy/lysosomal genes. Regarding neuronal TFEB overexpression, we were not able to detect any mRNA levels changes, probably due to a stronger neuronal cell death observed in this group (Figure 4).

**Figure 8:**
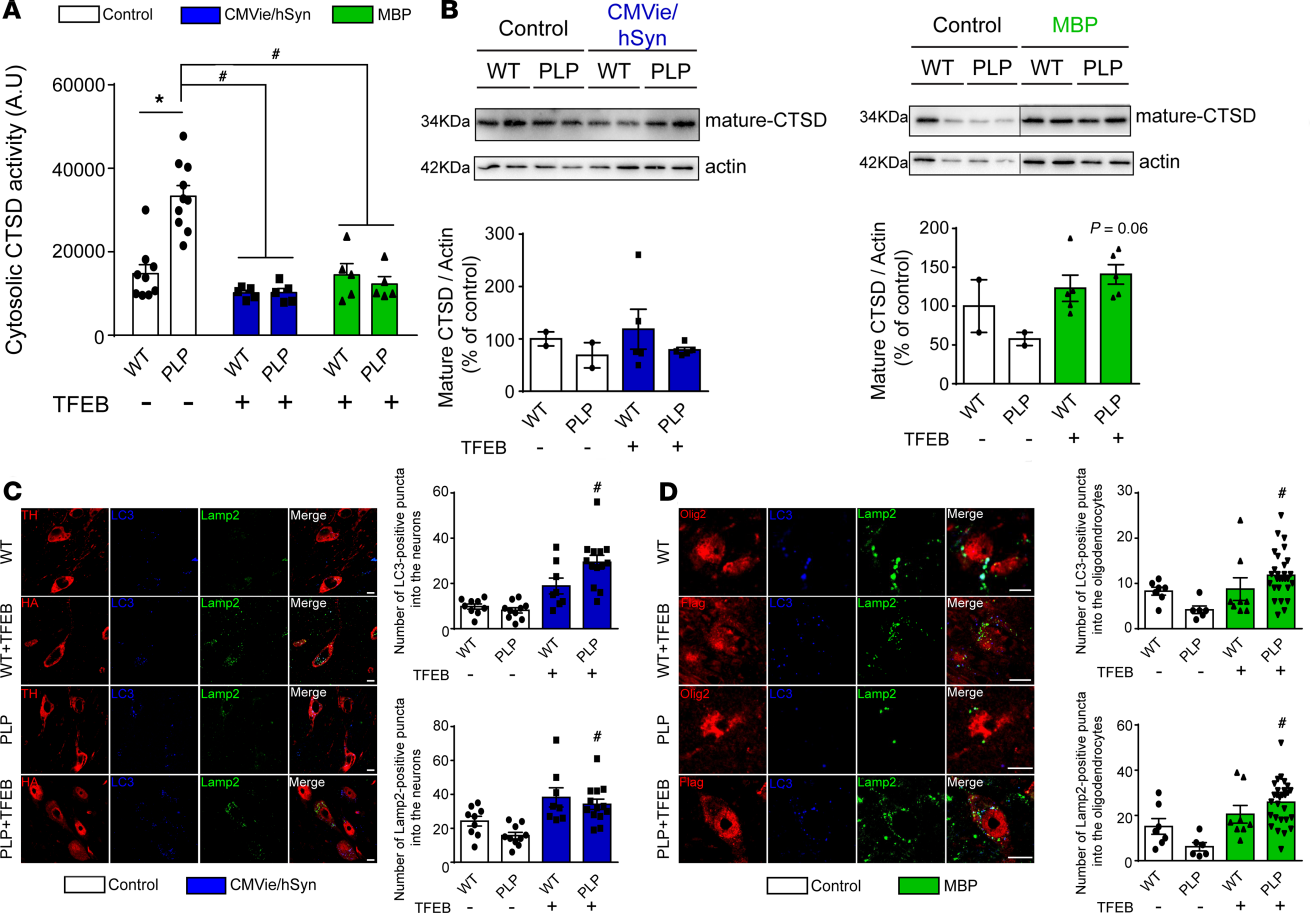
TFEB overexpression enhances autophagy-lysosomal pathway function in the brain of PLP mice. (**A**) Quantification of Cathepsin D (CTSD) activity in cytosolic lysosomal-free fraction from ipsilateral SN of control, CMVie/hSyn-mTFEB–injected, and MBP-mTFEB–injected WT and PLP mice. (**B**) CTSD immunoblot levels from ipsilateral SN of control, CMVie/hSyn-mTFEB–injected, and MBP-mTFEB- injected WT and PLP mice. *n* = 5 per group. Lanes were run on the same gel but were noncontiguous. (**C**) Confocal images (left) and quantification (right) using either TH or HA tag, Lamp-2, and LC3 antibodies in the ipsilateral SN of mice injected or not with the CMVie/hSyn-mTFEB-HA. The quantification represents the number of LC3- or Lamp-2–positive puncta into neuronal cells. Scale bar: 100 μm. *n* = 9–13 per group. (**D**) Confocal images (left) and quantification (right) using either Olig2 or Flag tag, Lamp-2, and LC3 antibodies in the ipsilateral SN of mice injected or not with the MBP-mTFEB-Flag. The quantification represents the number of LC3- or Lamp-2–positive puncta into oligodendrocytes. Scale bar: 50 μm. *n* = 7–30 per group. White bars, control; blue bars, CMVie/hSyn-mTFEB-HA; green bars, MBP-mTFEB-3×Flag. Data represent mean ± SEM. Comparisons were made using 1-way ANOVA and Tukey’s correction for multiple comparisons. *P < 0.05 compared with control WT animals. ^#^*P* < 0.05 compared with control PLP animals.

Because alteration of lysosomal integrity, and lysosomal membrane permeabilization especially, have been described in synucleinopathies (38, 39), we next explored whether overexpression of TFEB could rescue lysosomal membrane permeabilization in PLP mice. Accordingly, we measured Cathepsin-D (CTSD) activity, one of the most abundant lysosomal proteases, in lysosome-free cytosolic fractions. Compared with WT mice, PLP mice displayed significantly increased lysosomal membrane permeabilization, as evidenced by ectopic release of CTSD into the cytoplasm (Figure 8A). Following TFEB overexpression in either neurons or oligodendrocytes, cytosolic CTSD activity was fully restored to basal level of WT mice, suggesting either a protection of the lysosomal membrane integrity or enhancement of lysosomal biogenesis (Figure 8A). Further supporting a beneficial role on the lysosomal machinery, TFEB-injected PLP mice exhibited an increase in the mature form of CTSD expression levels in the SN compared with control PLP animals (Figure 8B). Similar results were obtained in mouse SN sections by immunostaining of the autophagosomal marker (LC3) and lysosomal-associated membrane protein 2 (LAMP-2) (Figure 8, C and D). We observed that the number of LC3- and LAMP-2–positive puncta in transduced dopaminergic neurons (Figure 8C; LC3, control vs. CMVie/hSyn-mTFEB PLP mice, F_[3,36]_ = 16.75, *P* < 0.0001; LAMP-2, control vs. CMVie/hSyn-mTFEB PLP mice, F_[3,47]_ = 8.77, *P* = 0.001) and in transduced oligodendrocytes (Figure 8D; LC3, control vs. MBP-mTFEB PLP mice, F_[3,47]_ = 4.71, *P* = 0.005; LAMP-2, control vs. MBP-mTFEB PLP mice, F_[3,36]_ = 8.49, *P* = 0.0001) in PLP mice was significantly increased compared with control PLP mice. Taken together, these data indicated that TFEB overexpression in neurons or oligodendrocytes increased autophagy flux through the formation of autophagosomes and lysosomal biogenesis, enhancing cellular clearance compared with the control group.

## Discussion

Here, we demonstrate that targeting neuronal expression of TFEB was sufficient to reduce synucleinopathy and prevent neurodegeneration in the A53T–α-syn rat model of PD, while only oligodendroglial overexpression of TFEB leads to neuroprotective effects in the MSA mouse model (Supplemental Figure 5). These beneficial effects were associated with a decrease of the pathological burden of α-syn through recovery of the ALP machinery. Overall, our study supports the idea that the cellular origin of the synuclein pathology dictates where enhancement of ALP should occur to allow a neuroprotective effect.

Numerous studies highlight lysosomal impairment as a key player in the pathogenesis of synucleinopathies such as PD and MSA (40, 41), and several therapeutic strategies based on ALP component overexpression, such as LAMP-2A (42) or Beclin-1 (43), have been used to increase autophagy machinery in experimental models of neurodegenerative diseases (44). Consistent with this approach, viral-mediated neuronal overexpression of TFEB has been used to mediate neuroprotection in a rat model of PD-overexpressing human WT α-syn (23). We here provide further evidence in another experimental model of PD. Neuronal expression of TFEB in the human mutated A53T–α-syn rat model of PD showed nigrostriatal dopaminergic neuroprotection and preserved motor function. Those beneficial effects were associated with decreased accumulation of α-syn.

However, no studies were performed so far to elucidate the role of the ALP in MSA pathogenesis and to exploit TFEB as a therapeutic target for this peculiar synucleinopathy. Here, we show that nuclear levels of TFEB are decreased in brains from patients with MSA and address the feasibility and therapeutic potential of restoring TFEB levels in selected cell types in a clinically relevant mouse model of MSA. Because MSA is first regarded as an oligodendrogliopathy, we tested whether TFEB overexpression either in neurons or in oligodendrocytes of the SN of PLP mouse model may result in different biological responses and alleviate MSA-related pathology. We here show that AAV-mediated expression of TFEB was sustainable in our model, and we observed nucleus localization of exogenous TFEB in neuronal cells or oligodendrocytes, which is necessary for its transcriptional role. Contrary to our rat model of PD, neuronal-targeted TFEB in PLP mice did not afford dopaminergic neuroprotection, reduction of synucleinopathy, or a decrease in inflammatory responses. This lack of efficacy of neuronal expression of TFEB in a MSA model could be due to the fact that, similar to the disease (45), neurons are less affected by the synucleinopathy compared with oligodendrocytes. Surprisingly, neuronal expression of TFEB in both WT and PLP mice showed potent neurotrophic effects, possibly linked to modulation of growth machinery, such as demonstrated in oncology (46), which should be further elucidated in future studies.

Importantly, we show that only specific oligodendroglial overexpression of TFEB in the PLP mice is able to partially overcome the deleterious effects associated with this model. According to our central hypothesis, overexpression of TFEB was effective in reversing the dopaminergic neurodegeneration in a higher magnitude at the level of striatal dopaminergic terminals compared with SN dopaminergic cell bodies through alleviation of the burden of aggregated α-syn. Notably, we confirmed that oligodendroglial-targeted TFEB overexpression also restores ALP function, attenuates lysosomal membrane permeabilization, and induces neurotrophic effects. Interestingly, the Akt pathway is involved in the process of myelination in the CNS (47), a role dedicated to the oligodendrocytes (48). This indicates that TFEB induces the Akt prosurvival pathways, which may help to reduce oligodendroglial dysfunction and lead to a more efficient myelin production, participating in dopaminergic pathway neuroprotection. Of course, future investigations will be necessary to determine its exact contribution and whether other prosurvival pathways are involved in the process of myelination, such as the TFEB gene target *MAPK1/3* (49).

Besides macroautophagy, cellular pathways such as CMA and UPS are involved in the degradation of α-syn, including in a MSA context (5, 50). Although enhancing ALP appears clearly efficient for mitigating α-syn accumulation in order to achieve neuroprotection, it could be relevant in future studies to combine complementary strategies through gene therapy or pharmacological drugs to enhance CMA and/or the UPS systems, in addition to TFEB for further optimizing α-syn clearance in the oligodendrocytes, in an attempt to reach even stronger neuroprotective effects.

In summary, the present study validates TFEB as an interesting therapeutic strategy in PD in an additional PD rat model, which is consistent with previous reports (23, 26, 51). Further supporting this interest, we provide the first evidence to our knowledge that targeting TFEB in a cell-specific manner is crucial in MSA pathology — i.e., oligodendroglial-targeted TFEB, as opposed to neuronal-targeted TFEB, leads to neuroprotective and neurotrophic effects with improvement of α-syn clearance after activation of lysosomal biogenesis in a transgenic MSA mouse model. Overall, we confirmed the relevance of targeting TFEB expression to enhance ALP as a promising therapeutic approach for all synucleinopathies. TFEB has also been validated as an emerging therapeutic target to enhance lysosomal biogenesis and autophagy in different disorders — such as Alzheimer’s disease, in which its astrocytic expression decreases misfolded Tau spreading (52) — but also for lysosomal storage disorders, such as Pompe Disease (53), as well as for ischemic injury (54), alcoholic liver disease (55, 56), and osteoarthritis (57). Recent reports in which the FDA-approved drug 2-Hydroxypropyl-β-cyclodextrin (24, 25) or the natural compound pomegranate extract (58) are TFEB expression enhancers pave the way for future intervention.

In conclusion, increasing TFEB expression, by gene therapy or through pharmacological activation, into the CNS but also into peripheral organs becomes a strongly relevant therapeutic strategy. Further investigations to address the extent and localization of TFEB induction are still fundamental to provide the most clinically relevant and safest candidate and therapeutic strategy.

## Methods

### Plasmid production

#### Rat study.

An AAV plasmid backbone containing the murine Tfeb cDNA fused to 3 Flag epitopes under control of the CMV promoter was provided by TIGEM AAV Vector Core Facility.

#### Mouse study.

AAV containing the murine Tfeb cDNA fused to HA epitope under control of the CMVie/hSyn and an AAV containing the murine Tfeb cDNA fused to 3 Flag epitopes under control of the MBP promoter were cloned in our laboratory.

### In vitro experiments

#### Assessment of Tfeb transgene expression.

Human embryonic kidney 293 cells (HEK293T) were obtained from ATCC (catalog CRL-11268) and grown in DMEM Low Glucose (MilliporeSigma) plus 10% FBS. HEK293T cells were plated into 12-well plates before being transfected with the mTFEB-containing plasmids at 1.6μg DNA using Polyethylenimine-mediated (PEI-mediated) transfection for 4 hours. Then, medium was changed, and cells were maintained for 48 hours at 37°C in 5% CO_2_ before being scraped into PBS. After a centrifugation step at 837 *g* for 5 minutes, the supernatant was removed, and cell pellet was lysed in 100 μl of Laemmli buffer (Tris-HCl 25 mM, pH 6.8, glycerol 7.5%, SDS 1%, DTT 250 mM, and Bromophenol blue 0.05% [MilliporeSigma]) for biochemical experiment.

#### Analysis of dendritic length.

Human neuroblastoma cell line, BE(2)-M17, obtained from ATCC (catalog CRL-2267) was cultured in OPTIMEM (Thermo Fisher Scientific) plus 10% FBS supplemented with 1% streptomycin/penicillin (MilliporeSigma). M17 were plated on coverslips into a 12-well plates to approximatively 80% confluency before being treated with retinoic acid (MilliporeSigma) at 5 μM for 24 hours (36). Cells were then transfected with the mTFEB-containing plasmids at 1.6 μg DNA using PEI-mediated transfection for 4 hours. Then, medium was changed, and cells were maintained for 48 hours at 37°C in 5% CO_2_ before being fixed with paraformaldehyde (VWR) at 4% for 30 minutes at 4°C for immunofluorescent staining. The fixed cells were washed 3 times with PBS 1× for 5minutes each time. Cells were then permeabilized with Triton 0.01% in PBS-NDS 3% for 30 minutes at room temperature before being incubated with the following primary antibodies (diluted in 1:1000): β3-tubulin (ab78078, Abcam) plus TFEB (Thermo Fisher Scientific, PA1-31552) overnight at 4°C. Cells were then washed 3 times with PBS 1× for 5 minutes each time before an incubation with donkey Alexa-conjugated antibodies (1:400, Invitrogen) in PBS. Cells were finally stained with DAPI solution (Invitrogen) at 10 μM for 8 minutes before long washes. Coverslips were mounted onto slides using mounting solution (Dako), and image acquisitions were made on a wide-field Zeiss Imager M2 and a CCD Camera Hamamatsu C10600 using Explora Nova MorphoStrider software. The dendritic length was measured using a segmentation analysis with the simple neurite tracer plugin of ImageJ (NIH).

### AAV vector production

Recombinant AAV9-CMVie/hSyn-mTFEB-HA-WPRE and AAV9-MBP-mTFEB-3×Flag-WPRE vectors were produced by PEI-mediated triple transfection of low-passage HEK-293T/17 cells (ATCC, catalog CRL-11268). The AAV expression plasmids were cotransfected with the adeno helper pAd Delta F6 plasmid (Penn Vector Core, catalog PL-F-PVADF6) and AAV Rep Cap pAAV2/9 plasmid (Penn Vector Core, catalog PL-T-PV008). AAV vectors were purified as previously described (59). Cells were harvested 72 hours after transfection, resuspended in lysis buffer (150 mM NaCl, 50 mM Tris-HCl, pH 8.5), and lysed by 3 freeze-thaw cycles (37°C/–80°C). The cell lysate was treated with 150 units/ml benzonase (MilliporeSigma) for 1 hour at 37°C, and the crude lysate was clarified by centrifugation. Vectors were purified by iodixanol step gradient centrifugation, and they were concentrated and buffer-exchanged into Lactated Ringer’s solution (Baxter) using vivaspin20 100 kDa cut-off concentrator (Sartorius Stedim). Titrations were performed at the platform study of the transcriptome (Neurocentre Magendie, INSERM U862). The genome-containing particle (gcp) titer was determined by quantitative PCR (qPCR) using the Light Cycler 480 SYBR green master mix (Roche Diagnostics) with primers specific for the AAV2 ITRs (forward, 5′-GGAACCCCTAGTGATGGAGTT-3′; reverse, 5′-CGGCCTCAGTGAGC GA-3′) (60) on a Light Cycler 480 instrument. Purity assessment of vector stocks was estimated by loading 10 μl of vector stock on 10% SDS acrylamide gels; total proteins were visualized using the Krypton Infrared Protein Stain according to the manufacturer’s instructions (Invitrogen). We obtained a titer of 3.06 × 10^12^ gcp/ml for the neuronal AAV9-CMVie/huSyn-mTFEB-HA-WPRE and a titer of 1.48 × 10^13^ gcp/ml for the oligodendroglial AAV9-MBP-mTFEB-3×Flag-WPRE.

### Rodent experiments and stereotactic inoculations

#### Rat study.

Thirty-two OFA Sprague Dawley rats (male, 2 months old) were injected unilaterally in the SNpc with 2 μl of either the AAV-A53Tα-syn (3.0 × 10^12^ vg/ml), the AAV-TFEB (3.0 × 10^12^ vg/ml), or a 1:1 mixture of AAV-A53Tα-syn/AAV-TFEB. Under isoflurane anesthesia, rats were placed in a stereotaxic frame (Kopf Instruments) and received 1 unilateral intranigral injections — either first track (–4.9 AP, 2.2 L, and –7.8 DV) or second track (coordinates from bregma: –5.1 antero-posterior [AP], 2 lateral [L], and –7.8 dorso-ventral [DV]) of either vector, as previously described in refs. 27 and 61.

#### Mouse study.

Homozygous transgenic PLP–α-syn mice (MGI:3,604,008) overexpressing human α-syn under the PLP (32) and background-, age-, and sex-matched nontransgenic C57BL/6J mice were used in this study (WT mice) — male and female mixed (bred in house). PLP mice were fully backcrossed onto C57BL/6J background (*n* > 15 generations). They were bred and housed in a temperature-controlled room under a 12/12 hours dark/light cycle, with free access to food and water.

PLP and WT mice (2 months old) received 2 μl of either AAV9-CMVie/huSyn-mTFEB-HA-WPRE or AAV9-MBP-mTFEB-Flag-WPRE virus (concentration: 3.06 × 10^12^ gcp/ml) by stereotactic delivery to the region immediately above the right SN (coordinates from bregma: AP, –2.9, L, –1,3, DV, –4.5) at a flow rate of 0.4 μl/min, and the pipette was left in place for 5 minutes after injection to avoid leakage. Animals were euthanized after 5 months. Ten mice were used in each group — male and female mixed. Five brains of each group were immediately freshly frozen by immersion in a cold isopentane bath at –60°C during 5 minutes and stored at –80°C for biochemistry investigation. The 5 others were postfixed for 3 days in 10 ml of 4% paraformaldehyde at 4°C, cryoprotected in gradient 20% sucrose in PBS before being frozen by immersion in a cold isopentane bath (–60°C) for at least 5 minutes, and stored immediately at –80°C until sectioning for histochemical analysis.

### mRNA extraction and qPCR

Nigral samples were homogenized in Tri-reagent (Euromedex), and RNA was isolated using a standard chloroform/isopropanol protocol (62). RNA was processed and analyzed following an adaptation of published methods (63). cDNA was synthesized from 2 μg of total RNA using RevertAid Premium Reverse Transcriptase and primed with oligo-dT primers and random primers (Fermentas). qPCR was performed using a LightCycler 480 Real-Time PCR System (Roche Diagnostics). qPCR reactions were done in duplicate for each sample, using transcript-specific primers, cDNA (4 ng), and LightCycler 480 SYBR Green Master (Roche Diagnotstics) in a final volume of 10 μl. The PCR data were exported and analyzed in an informatics tool (Gene Expression Analysis Software Environment) developed at the NeuroCentre Magendie. For the determination of the reference gene, the Genorm method was used (64). Relative expression analysis was corrected for PCR efficiency and normalized against 2 reference genes. The valosin containing protein (Vcp) and the hypoxanthine guanine phosphoribosyl transferase (Hprt) genes were used as reference genes. The relative level of expression was calculated using the comparative (2^–ΔΔCT^) method (64).

Primer sequences: Vcp (NM_009503) forward, 5′-TGGCCGTCTAGATCAGCTCAT-3′; Vcp (NM_009503) reverse, 5′-TTTCGCAGATTGGCTTTTAGG-3′; Hprt (NM_013556) forward, 5′-AAACAATGCAAACTTTGCTTTCC-3′; Hprt (NM_013556) reverse, 5′-CGAGAGGTCCTTTTC ACCAGC-3′; Mcoln1 (NM_053177) forward, 5′-TTCCTGCTGCAGAACGAGTTT-3′; Mcoln1 (NM_053177) reverse, 5′-CGTTCCCAGAGGCTG ATTTC-3′; Map1lc3a (NM_025735) forward, 5′-ACACCCATCGCTGACATCTATG-3′; Map1lc3a (NM_025735) reverse, 5′-TGGGAGGCGTAGACCATGTAG-3′; Lamp-2b (NM_010685) forward, 5′-AGATAATTGCTAGGCAGTGCCAA; Lamp-2b (NM_010685) reverse, 5′-GCTGCATGTAGA GGCCAATTTC-3′; CtsB (NM_007798) forward, 5′-ATGAGTGCCAGGCCTTTGAATA-3′; CtsB (NM_007798) reverse, 5′-GGCCATCGCCCAAATCTAT-3′; CtsF (NM_019861) forward, 5′-GGGC AAGAACCTGGCTACAGTAT-3′; CtsF (NM_019861) reverse, 5′-GCCTGCTGAGGACAGATC TAGTTT-3′.

### Biochemical analysis

#### Total protein extraction and quantification of mice tissue.

Tissue patches (*n* = 2–3) of mouse SN were extracted on ice using 80 μl of RIPA buffer (50 mM Tris-HCl pH 7.4, 150 mM NaCl, 1.0% Triton X-100, 0.5% Na-deoxycholate, 0.1% sodium dodecyl sulfate) with a protease inhibitor cocktail tablet (Complete Mini, Roche Diagnostics). The lysate was incubated on ice for 20 minutes, centrifuged at 18,220 *g* for 15 minutes at 4°C. The supernatant was collected, and the Bicinchoninic Acid (BCA) Assay (Thermo Fisher Scientific, USA) was used to determine the total amount of protein in the lysates and was then stored at –80°C. Based on total protein concentrations calculated from the BCA assays, aliquots of tissue lysates corresponding to known amounts of total protein per well were prepared for each animal in Laemmli buffer (Tris-HCl 25mM pH = 6.8, Glycerol 7.5%, SDS 1%, DTT 250mM and Bromophenol blue 0.05%) for the immunoblotting experiment.

#### Total protein extraction and quantification of human brain tissue.

Human putamen and frontal cortex were dissected from fresh frozen postmortem midbrain samples from 7 patients with MSA and 6 healthy controls (mean age at death: 72 ± 2.83 years; frozen postmortem interval: 27.5 ± 6.65 hours; GIE Neuro-CEB BB-0033-00011). For human brain samples of the putamen or the frontal cortex, we separated cytosolic and nuclear fractions by methods previously described (28). Proteins were extracted on ice using 1 ml of buffer A (10 mM HEPES, pH 7.9, 10 mM NaCl, 0.1 mM EDTA, and 1 mM dithiothreitol) with a protease inhibitor cocktail tablet (Complete Mini, Roche Diagnostics). The homogenate was centrifuged at 837 *g* for 5 minutes at 4°C. The supernatant was collected as cytosolic fractions. For nuclear fractions, the pellet was dissolved in buffer B (20 mM HEPES, pH 7.9, 400 mM NaCl, 1 mM EDTA, and 1 mM dithiothreitol) with a protease inhibitor cocktail tablet (Complete Mini, Roche Diagnostics) and vortexed on ice for 15 minutes. The homogenate was incubated for 30 minutes at 4°C under constant shaking before a centrifugation at 18,220 *g* for 10 minutes at 4°C. Supernatant was collected as nuclear fractions. The BCA assay was used to determine the total amount of protein in the nuclear and cytosolic fractions and was then stored at –80°C. Based on total protein concentrations calculated from the BCA assays (Thermo Fisher Scientific), aliquots of tissue fractions corresponding to known amounts of total protein per well were prepared for each individual in Laemmli buffer (Tris-HCl 25mM pH = 6.8, Glycerol 7.5%, SDS 1%, DTT 250mM and Bromophenol blue 0.05%) for the immunoblotting experiment.

#### Immunoblotting analysis.

Western blots were run from 10 μg of protein extracts from mouse SN, 20 μl of transfected cell lysates, and 20 μg of protein extracts from brain patients, separated by SDS-PAGE and transferred to 0.2 μm nitrocellulose membrane (Bio-Rad). Incubation of the primary antibodies was performed overnight at 4°C with goat anti-mTFEB (1:1,000, Thermo Fisher Scientific, PA1-31552), rabbit anti-huTFEB (1:1,000, Cell Signaling Technologies, 4240), goat anti-HA (1:1,000, Genscript, A00168), mouse anti-Flag (1:1,000, MilliporeSigma, F3165), mouse anti–cath-D (1:1,000, MilliporeSigma, C0715), rabbit anti-Akt (1:1,000, Cell Signaling Technology, 9272), and rabbit anti–Ser473-phosphorylated-Akt (1:1,000, Cell Signaling Technology, 9271). Mouse anti-actin (1:2000, MilliporeSigma, A5441) was used to control equal loading except for nuclear fraction of brains patients (LaminA/C, 1:2,000, Genscript, A01455). Appropriate secondary antibodies coupled to peroxidase were revealed using a Super Signal West Pico Chemiluminescent kit (Immobilon Western, Chemiluminescent HRP substrate, MilliporeSigma). Chemiluminescence images were acquired using the ChemiDoc+XRS system measurement (Bio-Rad). Signals per lane were quantified using ImageJ, and a ratio of signal on loading per sample was performed and used in statistical analyses. Regarding biochemical experiments on mice tissue, each graph regarding immunoblotting experiments on mice represents the quantified protein level normalized by actin protein levels. Regarding TFEB protein levels for MSA brains, the calculated ratio nuclear TFEB/cytosolic TFEB corresponds to the ratio “nuclear TFEB/Lamin” divided by the ratio “cytosolic TFEB/Actin”. See complete unedited blots in the supplemental material.

#### CTSD activity assay.

CTSD activity was measured in cell lysates using a fluorometric CTSD activity assay kit (Abcam, ab65302) in accordance with the manufacturer’s instructions. Fluorescence was measured on a FLUOstar Optima microplate analyzer (BMG Labtech).

### Histopathological analysis

#### Extent of lesion.

To assess the integrity of the nigrostriatal pathway, TH IHC was performed on SNpc and striatal sections. Briefly, sections from 3 representative levels of the striatum (anterior, medial, and posterior) and serial sections (1 of 6) corresponding to the whole SNpc were incubated with a rabbit monoclonal antibody raised against mouse TH (Abcam, EP1532Y, ab137869, 1:5,000) for 1 night at room temperature and revealed by an anti–rabbit peroxidase EnVisionTM system (DAKO, K400311) followed by DAB visualization. Free-floating SNpc sections were mounted on gelatinized slides, counterstained with 0.1% cresyl violet solution, dehydrated, and cover-slipped, while striatal sections were mounted on gelatinized slides and cover-slipped. The extent of the lesion in the striatum was quantified by OD. Sections were scanned in an Epson expression 10000XL high-resolution scanner, and images were used in ImageJ open source software to compare the gray level in the putamen. TH-positive SNpc cells were counted by stereology, blind with regard to the experimental condition using a Leica DM6000B motorized microscope coupled with the Mercator software (Explora Nova). The SN was delineated for each slide, and probes for stereological counting were applied to the map obtained. Each TH-positive cell with its nucleus included in the probe was counted. The optical fractionator method was finally used to estimate the total number of TH-positive cells in the SNpc of each mouse hemisphere. In addition, we measured the Nissl cell count and the surface of TH occupied in SN to fully characterize the pattern of dopaminergic cell loss in the SN. The surface is an additional quantification method that we use in tissue sections. For these analyses, a specific staining process was used to keep all tissues together in the same solution during the staining process, counterstained with 0.1% cresyl violet solution. Then, high-resolution whole color slide images were first acquired with the 3D Histech Panoramic Scanner at the 20× magnification, with 5 layers in extended mode. Each image was opened in the off-line MERCATOR PRO 7.12.3 software (Explora Nova), and the mapping of all regions of interest was made. Brightness and contrast rules were applied to the RGB pictures to optimize details without any saturation of the image. The color thresholding tool was then used to select the threshold corresponding to the brown color revealed by the DAB staining. The threshold has been established on the basis of the staining intensity to detect the maximum of DAB staining. The file of the threshold parameters was saved and applied to all measurements for each animal/staining. Before performing the quantification, the threshold was randomly applied to some images of different treatment groups to verify the accuracy of settings. In each region, the software extracted the surface corresponding to the threshold defined. The surface parameter was finally expressed as a ratio of the total surface of each area of interest. Conversely, the stereology approach allowed us to obtain an unbiased number of cells, while the threshold surface analysis allowed us to quantify any antibody-based staining in the region of interest. Both, therefore, are needed.

#### α-Syn pathology.

Synucleinopathy has been assessed with a mouse monoclonal antibody raised against human α-syn (Novex, Invitrogen, LB509, 180215, 1:1,000) using the M.O.M. Vector kit protocol and against phosphorylated α-syn (Abcam, EP1536Y, ab51253, 1:5,000) immunostaining, as we previously reported (27, 65). Briefly, selected sections of 1 rostro-caudal level of SN and striatum were specifically identified and incubated in the same well to allow direct comparison of immunostaining intensity. For pretreatment with PK, sections were incubated first with PK at 10 μg/ml in PBS before long sequential washes in distilled water and then in PBS. Sections were incubated overnight at room temperature with the aforementioned antibodies. The following day, revelation was performed with anti-species peroxidase EnVision system (DAKO) followed by DAB incubation. Sections were then mounted on gelatinized slides, dehydrated, counter-stained if necessary, and cover-slipped until further analysis. LB509-positive dots in SN were counted by stereology, blind with regard to the experimental condition using a Leica DM6000B motorized microscope coupled with the Mercator software (Explora Nova). The SN was delineated for each slide, and probes for stereological counting were applied to the map obtained (size of probes was 60 × 80 μm) and transform as object after counting. The optical fractionator method was finally used to estimate the total number of LB509-positive dots per μm² in the SN of each mouse. LB509 immunostaining–positive surface in the striatum and phosphorylated α-syn immunostaining–positive surface quantification in the SN and the striatum were performed as previously (27).

#### Inflammation.

Inflammatory processes in the SN and the striatum were measured through GFAP/S-100 (DAKO, Z0334/Abnova, PAP11341) and Iba1 (Abcam, ab5076) IHC. Striatal sections of all animals were incubated together overnight with a mix of rabbit antibodies raised against GFAP and S-100 for the astroglial staining (respective dilutions 1:2,000 and 1:1,000) and with a rabbit anti-Iba1 antibody for the microglial staining (dilution 1:1,000). These signals were revealed with anti-species peroxidase EnVision system (DAKO) followed by DAB incubation. Sections were mounted on slides, dehydrated, and cover-slipped. Sections were scanned in a high-resolution scanner (PanScan, 3D Histech) at ×20 magnification, and the quantification of GFAP-positive astrocytic reaction or Iba1-positive microglial reaction was estimated by an immunostaining-positive surface quantification at regional levels with the Mercator software (Explora Nova).

### Immunofluorescent images

#### For 2′,3′-cyclic nucleotide-3′-phosphodiesterase (CNPase) and Flag fluorescent costaining.

For CNPase staining, tyramide signal amplification (TSA) protocol kit was used. Briefly, sections were permeabilized for 30 minutes in TSA blocking buffer containing 0.1% Tween20 (MilliporeSigma) and incubated overnight at room temperature with mouse anti-CNPase (Abcam, ab6319, 1:1,000) primary antibody diluted in a 1% goat serum/PBS buffer. Sections were then washed with PBS 3 times for 10 minutes and then incubated for 10 minutes in PBS/H_2_O_2_ 3%. After washing, sections were incubated for 2 hours with a goat biotinylated anti-mouse (Vector Laboratories, BA-9200) (1:200) at room temperature. Sections were then washed 3 times in PBS before being blocked with a streptavidin HRP diluted in TSA blocking solution (1:100) for 30 minutes. After an amplification step with Biotinyl tyramide diluted in amplification diluent followed by 3 washes in PBS, sections were incubated with goat anti–mouse biotinylated IgG conjugated to AlexaFluor probe 488 (Invitrogen, 1:1000) for 30minutes. Sections were then washed and incubated with rabbit anti-Flag (MilliporeSigma, F7425, 1:500) overnight before 3 steps of wash. Then, sections were incubated with goat anti–rabbit IgG conjugated to AlexaFluor 568 (Invitrogen, 1:1,000) for 1.5 hours in PBS. Tissues were then washed with PBS and mounted in DAPI-containing mounting media (Vectashield). Illustrative images were acquired using a confocal microscope Leica SP8 at the BioImaging Center in Bordeaux.

#### HA and TH fluorescent costaining.

Sections were permeabilized for 1 hour in a 4% donkey serum/PBS blocking buffer containing 0,3% Triton X-100 (MilliporeSigma) and incubated overnight at 4°C with the following primary antibodies diluted in a 1% Donkey serum/PBS buffer: rabbit anti-TH (Abcam, EP1532Y, ab137869, 1:2,000) and goat anti-HA (Genscript, A00168, 1:500). Following incubation with primary antibodies, tissues were washed with PBS 3 times for 10 minutes and incubated for 1.5 hours at room temperature with a combination of corresponding donkey anti-species IgG conjugated to AlexaFluor probe (Invitrogen, 1:400). Tissues were then washed with PBS and mounted in DAPI-containing mounting media (Vectashield). Illustrative images were acquired using a confocal microscope Leica SP8 at the BioImaging Center in Bordeaux.

#### Lamp2 and LC3 staining.

Sections were permeabilized for 1 hour in a 4% donkey serum/PBS blocking buffer containing 0.3% Triton X-100 (MilliporeSigma) and incubated overnight at 4°C with the following primary antibodies diluted in a 1% donkey serum/PBS buffer: mouse anti-TH (Merck, MAB318, 1:2,000), goat anti-HA (Genscript, A00168, 1:500), mouse anti-Olig2 (Merck, MABN50, 1:500), mouse anti-Flag (1:1,000, MilliporeSigma, F3165, 1:500), rat anti–Lamp-2 (Abcam, Abl93, ab25339, 1:1,000), and rabbit anti-LC3 (Novus Biological, NB2220, 1:1,000). Following incubation with primary antibodies, tissues were washed with PBS 3 times for 10 minutes and incubated for 1.5 hours at room temperature with a combination of corresponding donkey anti-species IgG conjugated to AlexaFluor probe (Invitrogen, 1:400). Tissues were then washed with PBS and mounted in DAPI-containing mounting media (Vectashield). Illustrative images were acquired using a confocal microscope Leica SP8 at the BioImaging Center in Bordeaux.

### Statistics

Statistical analyses were performed with GraphPad Prism 6.0 (GraphPad Software Inc.). For rat experiments, comparisons among means were performed by using 1-way ANOVA followed, if appropriate, by a pairwise comparison between means by Tukey post hoc analysis. For in vivo mice experiments, comparisons among means were performed by using 2-way ANOVA followed, if appropriate, by a pairwise comparison between means by Tukey post hoc analysis. For in vitro experiments, comparisons among means were performed by using nonparametric 2-tailed *t* test or 1-way ANOVA followed, if appropriate, by a pairwise comparison between means by Tukey post hoc analysis. All values are expressed as the mean ± SEM. Each dot in the scatter plot represents 1 individual. In all analyses, statistical significance was set at *P* < 0.05. For Figure 7C, code was written using the Python scientific stack and plotted using Matplotlib (66–68).

### Study approval

#### Animals.

Experiments were performed in accordance with the European Union directive of September 22, 2010 (2010/63/EU), on the protection of animals used for scientific purposes. The Institutional Animal Care and Ethical Committee of Bordeaux University (CE50, France) approved experiments accepted by the ministry under reference APAFIS 9921-2017031014326763 v5.

#### Human tissues.

Samples were obtained from brains collected in a Brain Donation Program of the Brain Bank GIE NeuroCEB run by a consortium of Patient Associations: association for research on multiple sclerosis (ARSEP), cerebellar ataxias (CSC), France Alzheimer, and France Parkinson. The consents were signed by the patients themselves or their next of kin in their name, in accordance with the French Bioethical Laws. The Brain Bank GIE NeuroCEB (Bioresource Research Impact Factor number BB-0033-00011) has been declared at the Ministry of Higher Education and Research and has received approval to distribute samples (agreement AC-2013-1887).

## Author contributions

MLA, MB, BD, and EB conceived and designed the study. MB performed all the surgeries, histochemical and immunofluorescent experiments and analysis on the A53T–α-syn PD rat model. MLA performed all the surgeries, along with histochemical, biochemical, and immunofluorescent experiments and analysis on the PLP mouse model of MSA. ND produced the viruses used in the study. MLT performed the patch extraction on mice tissues. ED participated in in vivo immunofluorescent experiments and confocal imaging acquisition. SD participated in immunohistochemical protocols and analysis for the MSA mouse model. AB provided the AAV plasmid backbone containing the murine Tfeb cDNA. POF worked as a referent for the PLP mouse model. WGM provided human brain tissues from MSA patients. MLA, MB, BD, SD, and EB analyzed the data. MLA, MB, BD, and EB wrote the paper. All authors discussed the results, assisted in the preparation, and contributed to the manuscript. All authors approved the final version of the manuscript.

## Supplementary Material

Supplemental data

## Version 1 08/22/2019

Electronic publication
