# Supplementary material for: Transcription factor EB overexpression prevents neurodegeneration in experimental synucleinopathies
Source: JCI Insight. 2019 Aug 22;4(16):e129719. doi: 10.1172/jci.insight.129719 (PMC6777809; doi:10.1172/jci.insight.129719)

Supplemental information

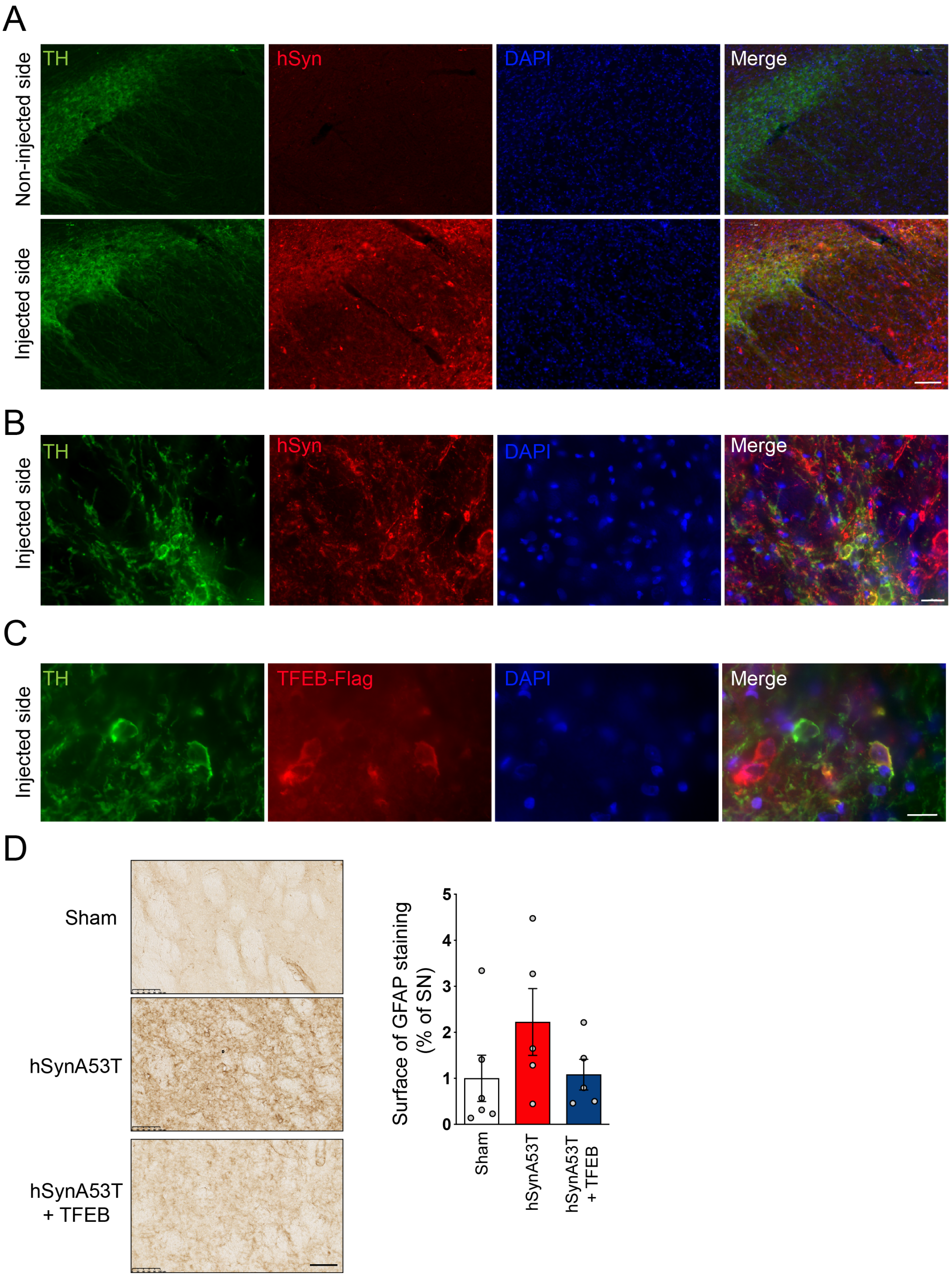

Supplementary Figure 1. Virus transduction and astrogliosis in the human A53T-α-syn rat model of Parkinson's disease. (A-B) AAV-delivered human A53T α-syn is expressed in TH-positive neurons in the SNpc as revealed by anti-human α-syn immunodetection. Scale bars = 100µm (A), 20µm (B). **(C)** AAV-mediated TFEB-3xFLAG is expressed in TH-positive neurons in the SNpc as revealed by anti-FLAG immunodetection. Scale bar = 20µm. **(D)** TFEB overexpression decreases astrogliosis associated with human A53T mutant α-syn overexpression in the striatum. Representative pictures and surface quantification of GFAP immunostaining. Scale bar = 100µm.

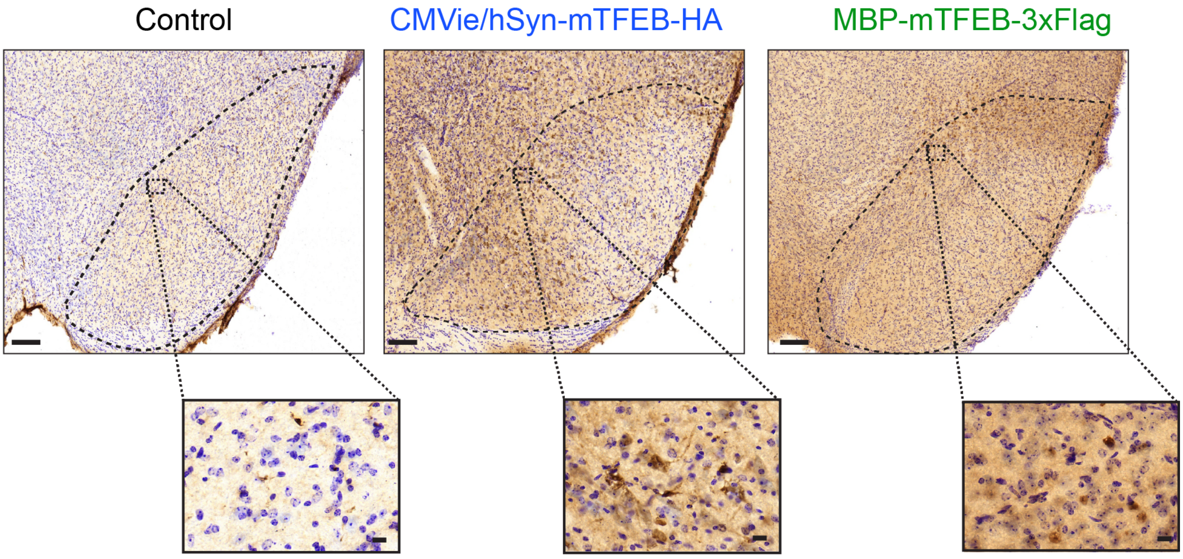

Supplementary Figure 2. Virus transduction in the Substantia Nigra of TFEB-injected mice. Representative images of virus transduction in the SN of control and TFEB-injected mice using immunostaining against HA tag for CMVie/hSyn-mTFEB group and Flag tag for MBP-mTFEB group. Scale bars = 200µm, 20µm (inset).

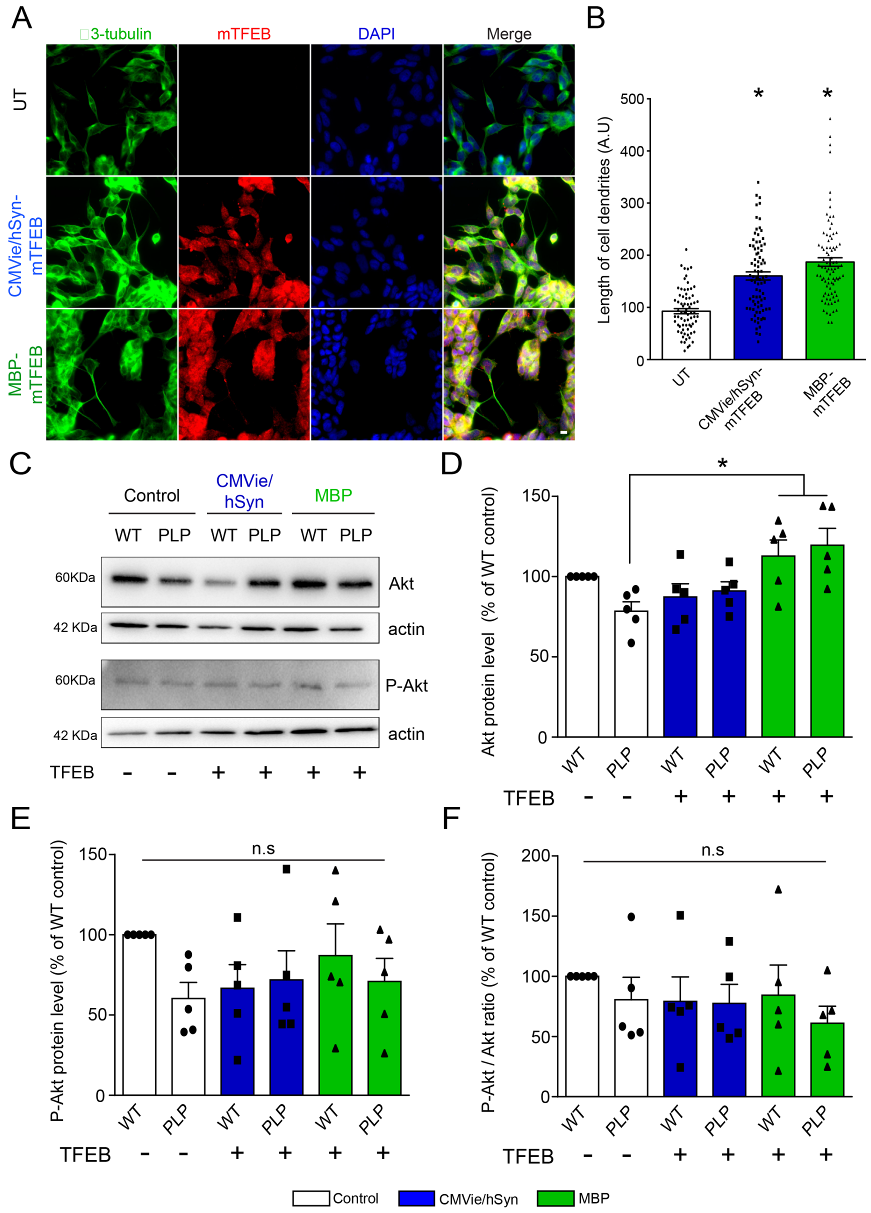

Supplementary Figure 3. TFEB-mediated neurotrophic effect *in vitro* in the dendritic length. (A) Illustrative images using β3-tubulin (green) and TFEB (red) antibodies in BE(2)-M17 human dopaminergic neuroblastoma cells treated with 5µM retinoic acid for 24h and transfected or not with TFEB-encoding plasmids for 48h. **(B)** Quantification of the dendritic length in non-transfected cells (UT, n= 71, *white bar*), CMVie/hSyn-mTFEB (n = 83, *blue bar*) and MBP-TFEB-transfected cells (n= 88, *green bar*) expressed in arbitrary unit (A.U). Scale bar = 10µm. (**C**) Representative images of Akt and phosphorylated Akt (P-Akt) immunoblotting in the ipsilateral SN of Control, CMVie/hSyn-mTFEB-injected and MBP-mTFEB- injected WT and PLP mice. **(D-E-F**) Quantification of Akt **(D)**, Phospho-Akt **(E)** and the ratio P-Akt/Akt **(F)** protein levels expressed in percentage of WT control mice in the ipsilateral SN of Control, CMV-mTFEB-injected and MBP-mTFEB-injected WT and PLP mice. n=5 per group. White bars: control; blue bars: CMVie/hSyn-mTFEB-HA; green bars: MBP-mTFEB-3xFlag. Data represent mean +/- SEM. In B, *c*omparisons were made using One-Way ANOVA and Tukey's correction for multiple comparisons. *p< 0.05 compared to control non-transfected cells. In D-E-F, comparisons were made using Two-Way ANOVA and Tukey's correction for multiple comparisons. *p< 0.05 compared to Control PLP animals. n.s: not significant.

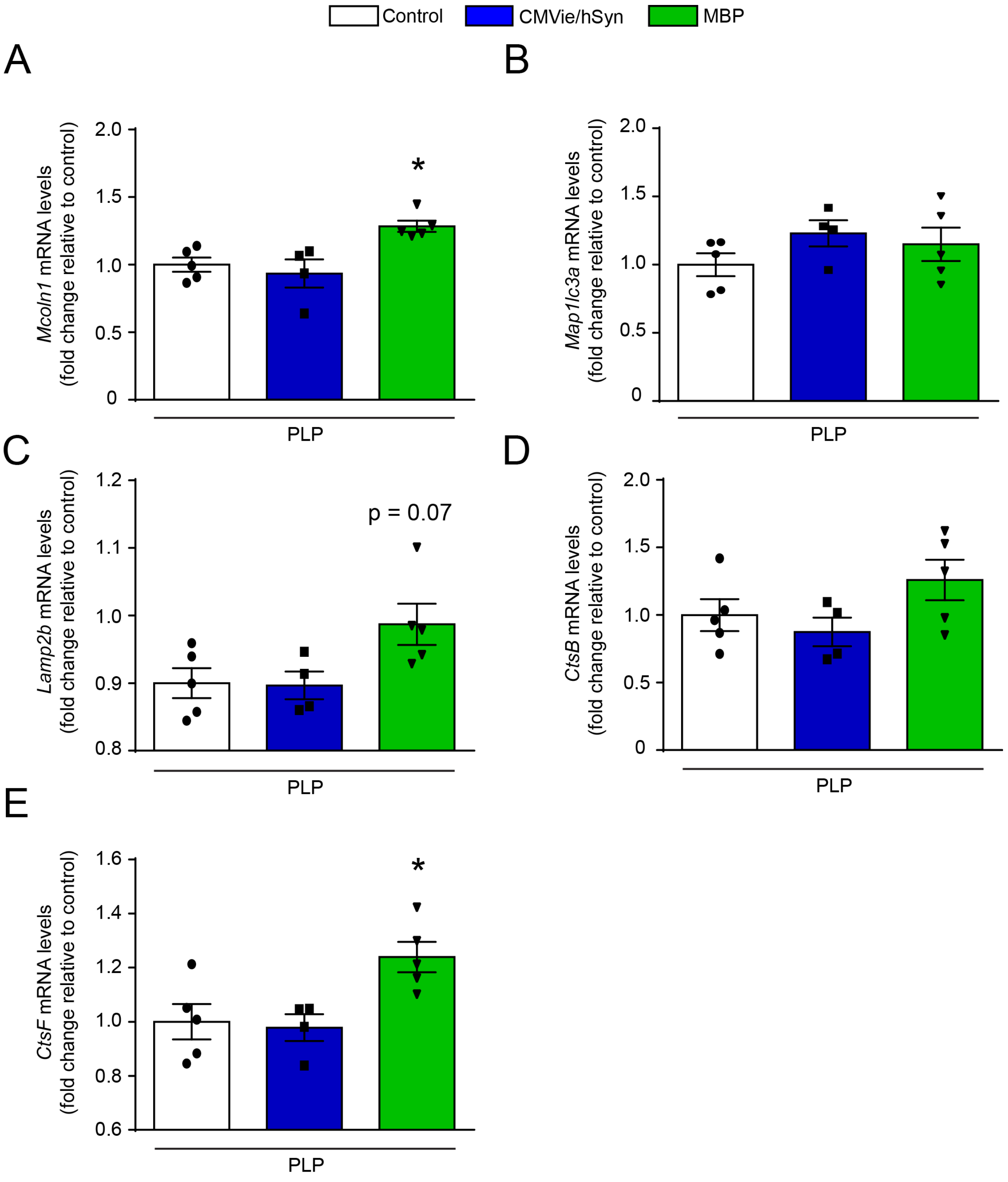

Supplementary Figure 4. Gene expression analysis of *Tfeb* lysosomal targets in the Substantia Nigra of TFEB-injected PLP mice. mRNA levels measured by quantitative PCR in SN patches of control and TFEB-injected PLP mice of *Mcoln1* **(A)**, *Map1lc3a* **(B)**, *Lamp2b* **(C)**, *CtsB* **(D)** and *CtsF* **(E)** genes. n=4-5 per group. White bars: Control; Blue bars: CMVie/hSyn-mTFEB-HA, Green bars: MBP-mTFEB-3xFlag. Data represent mean +/- SEM. Comparisons were made using One-Way ANOVA and Tukey's correction for multiple comparisons. *$p < 0.05$ compared to control PLP mice.

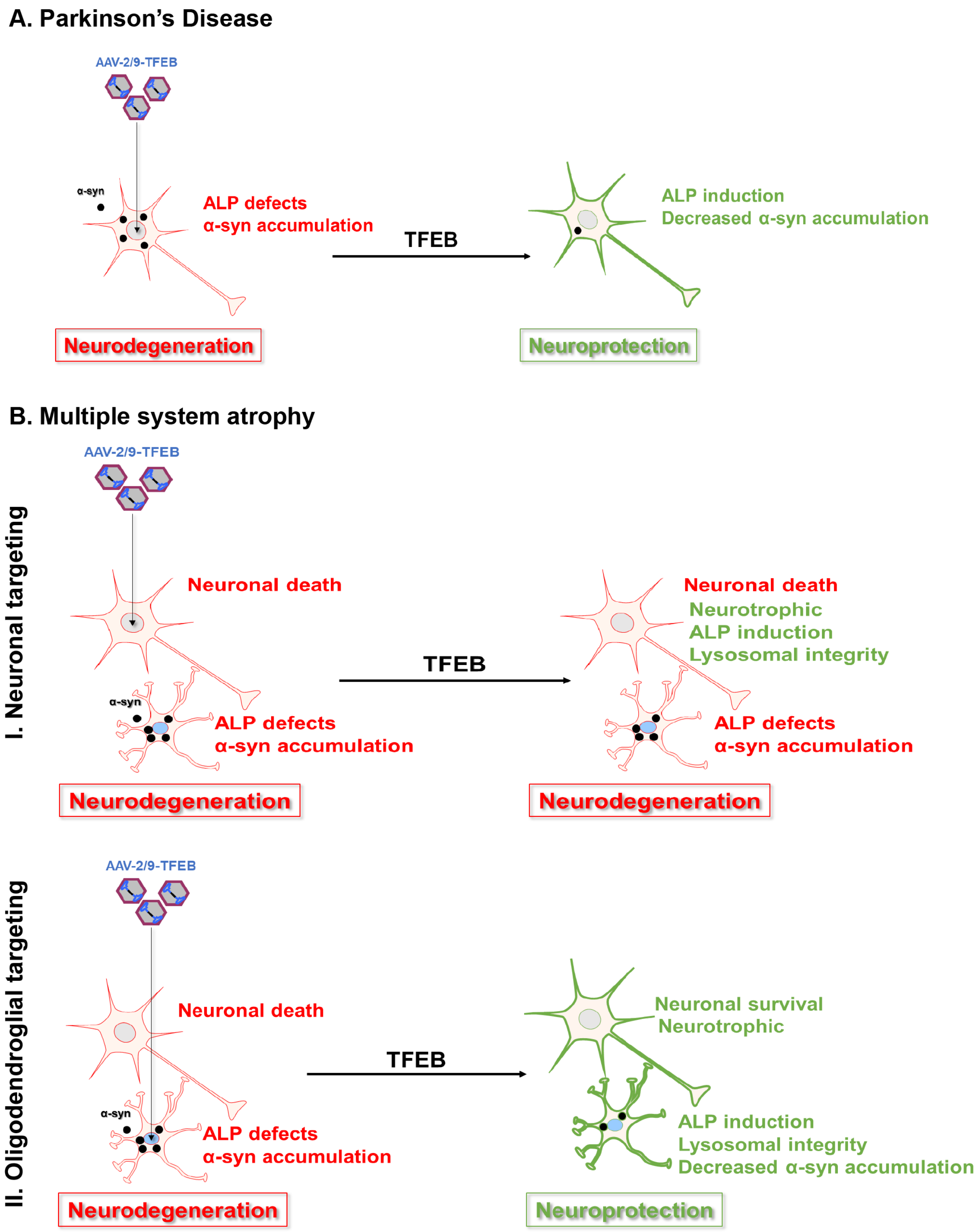

Supplementary Figure 5. Cell-specific targeting of TFEB overexpression as a therapeutic strategy to prevent neurodegeneration in synucleinopathies. (A) Overexpressing TFEB in dopaminergic neurons led to neuroprotective effects associated with decreased α-synuclein levels linked to ALP enhancement in a rat model of Parkinson's Disease**. (B) (I.)** Overexpressing TFEB in dopaminergic neurons induces neurotrophic effects but not neuroprotective effects in MSA mice model**. (II.)** Overexpressing TFEB in oligodendrocytes induces neurotrophic effects but also neuroprotective effects associated with decreased α-synuclein levels and ALP enhancement in a mice model of MSA.

Supplementary Table 1. Demographics of healthy control subjects and MSA patients obtained from GIE Neuro-CEB BB-0033-00011 used in this study. PMI: post-mortem interval, M: male, F: female, MSA-p: Multiple system atrophy, parkinsonian type.

Numbers	Diagnosis	Age (y)	Sex	PMI (h)	Tissue	
1	Normal control	84	M	15.5	Putamen	Frontal Cortex
2	Normal control	79	M	Nd	Putamen	Frontal Cortex
3	Normal control	69	M	6	Putamen	Frontal Cortex
4	Normal control	83	F	21	Putamen	Frontal Cortex
5	Normal control	80	M	4	Putamen	Frontal Cortex
6	Normal control	78	M	23	Putamen	Frontal Cortex
7	MSA-p	54	M	5.25	Putamen	Frontal Cortex
8	MSA-p	64	F	6.5	Putamen	Frontal Cortex
9	MSA-p	60	F	Nd	Putamen	Frontal Cortex
10	MSA-p	57	F	7	Putamen	Frontal Cortex
11	MSA-p	75	M	72	Putamen	Frontal Cortex
12	MSA-p	78	F	48	Putamen	Frontal Cortex
13	MSA-p	77	M	39	Putamen	Frontal Cortex

Full unedited gel for Figure 2A

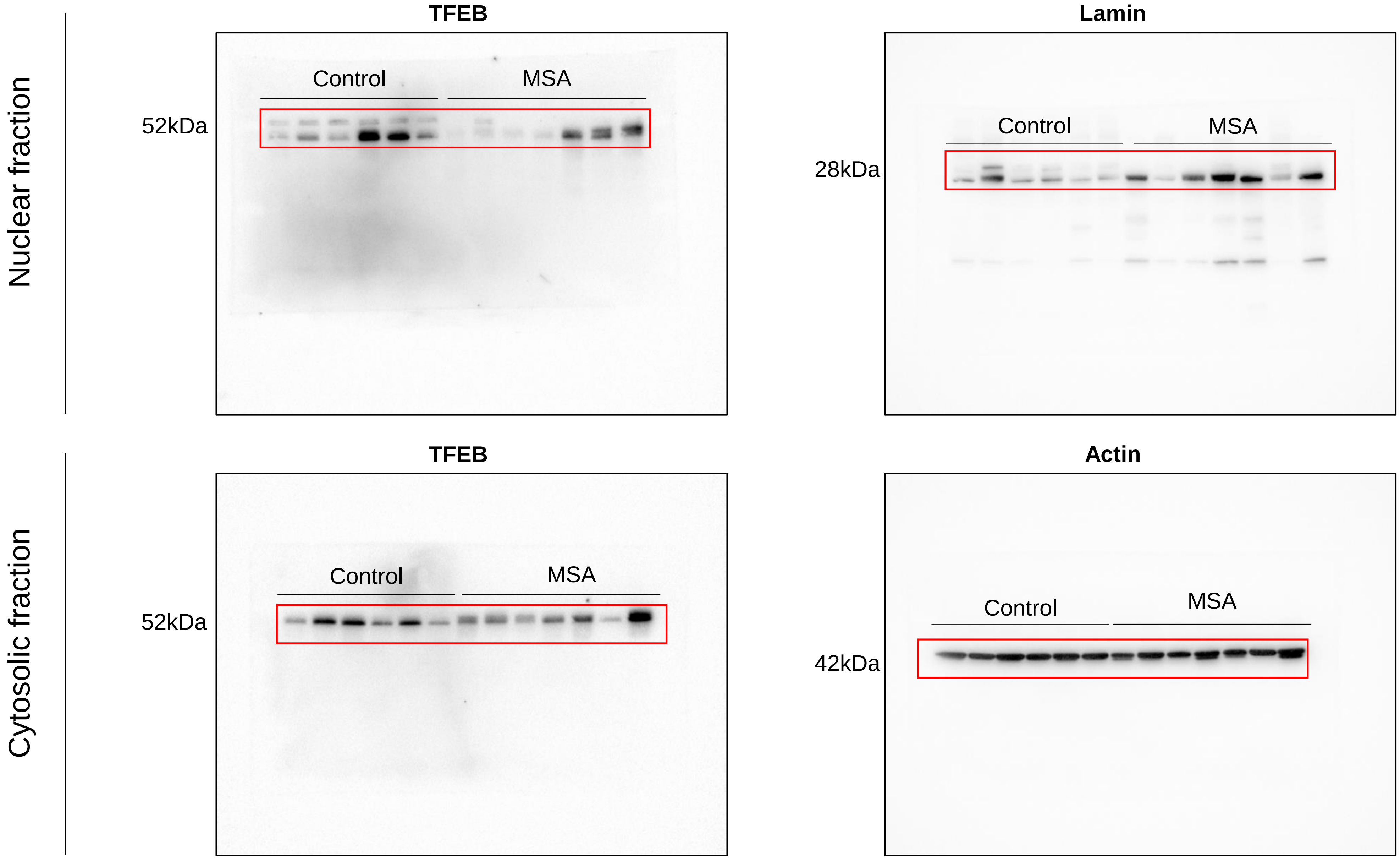

Full unedited gel for Figure 2C

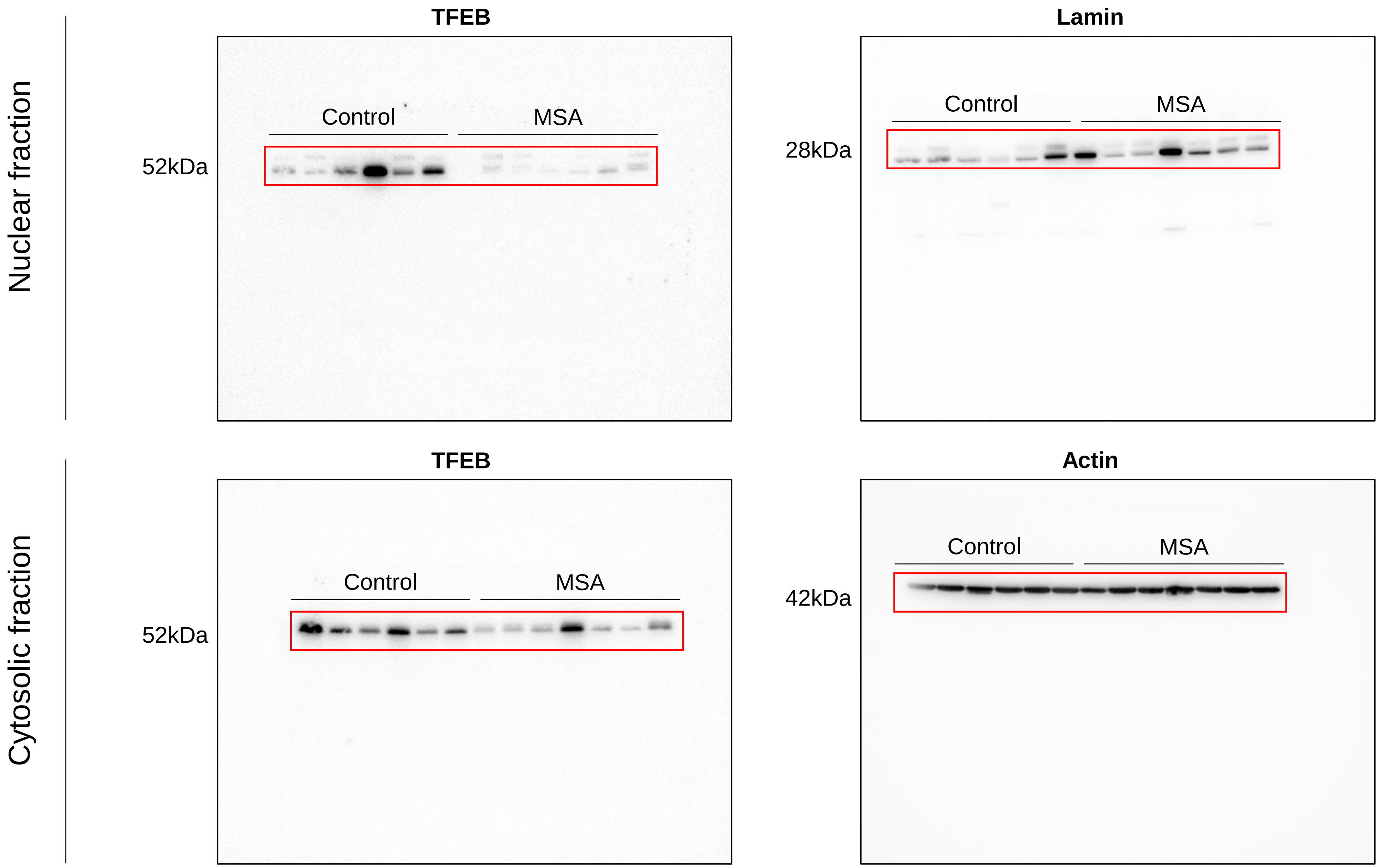

Full unedited gel for Figure 3A

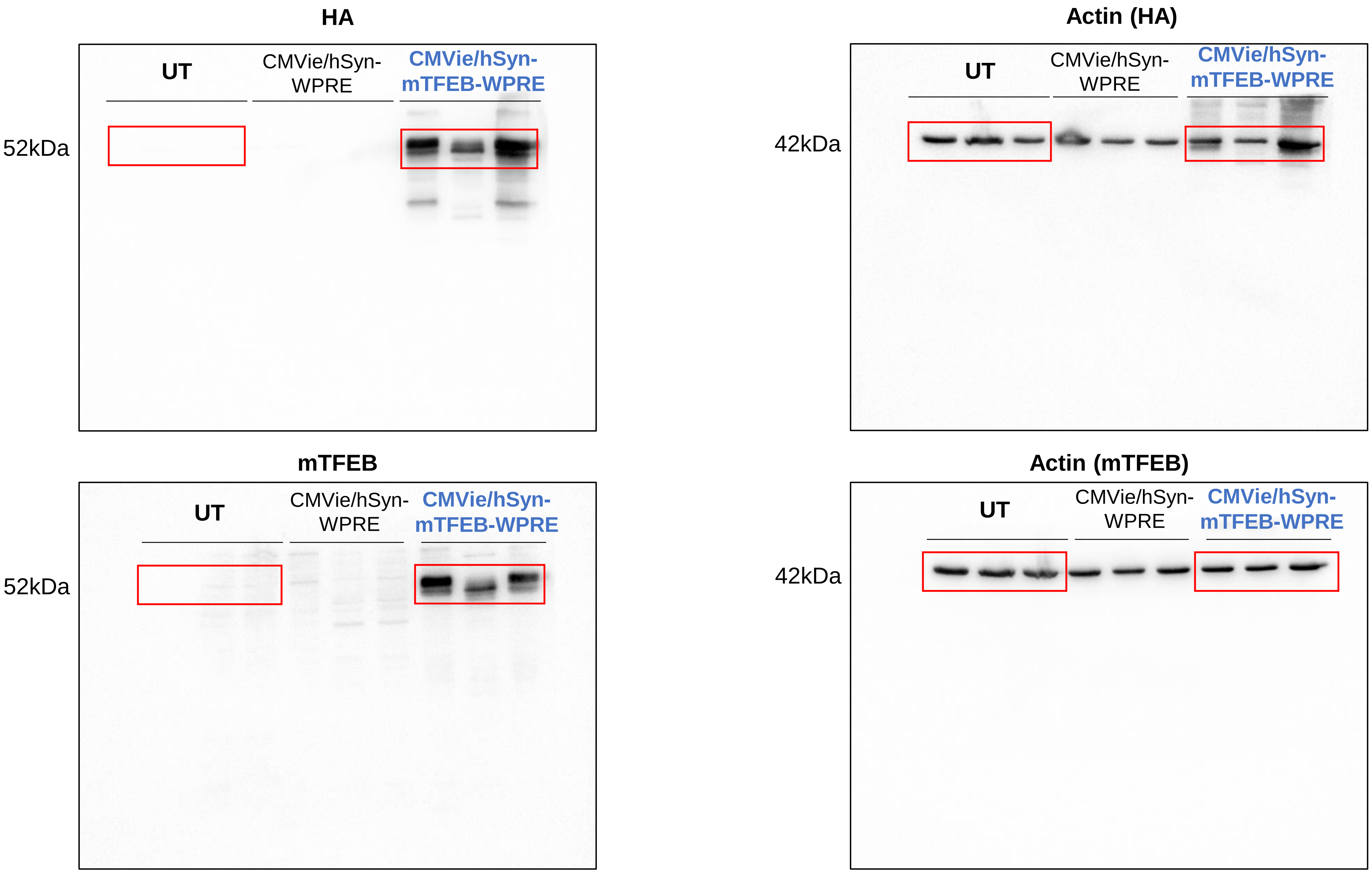

Full unedited gel for Figure 3B

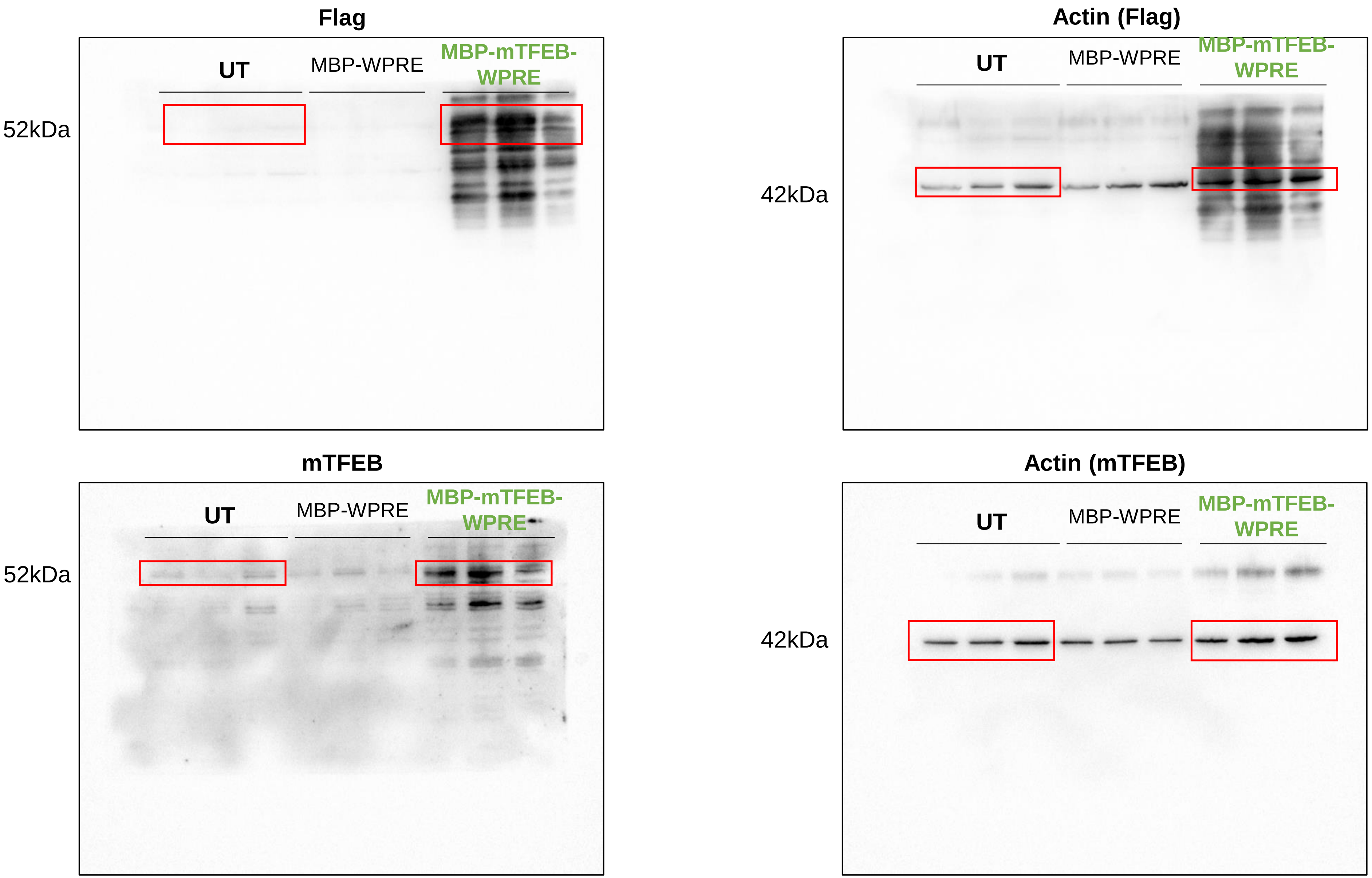

Full unedited gel for Figure 3D

HA

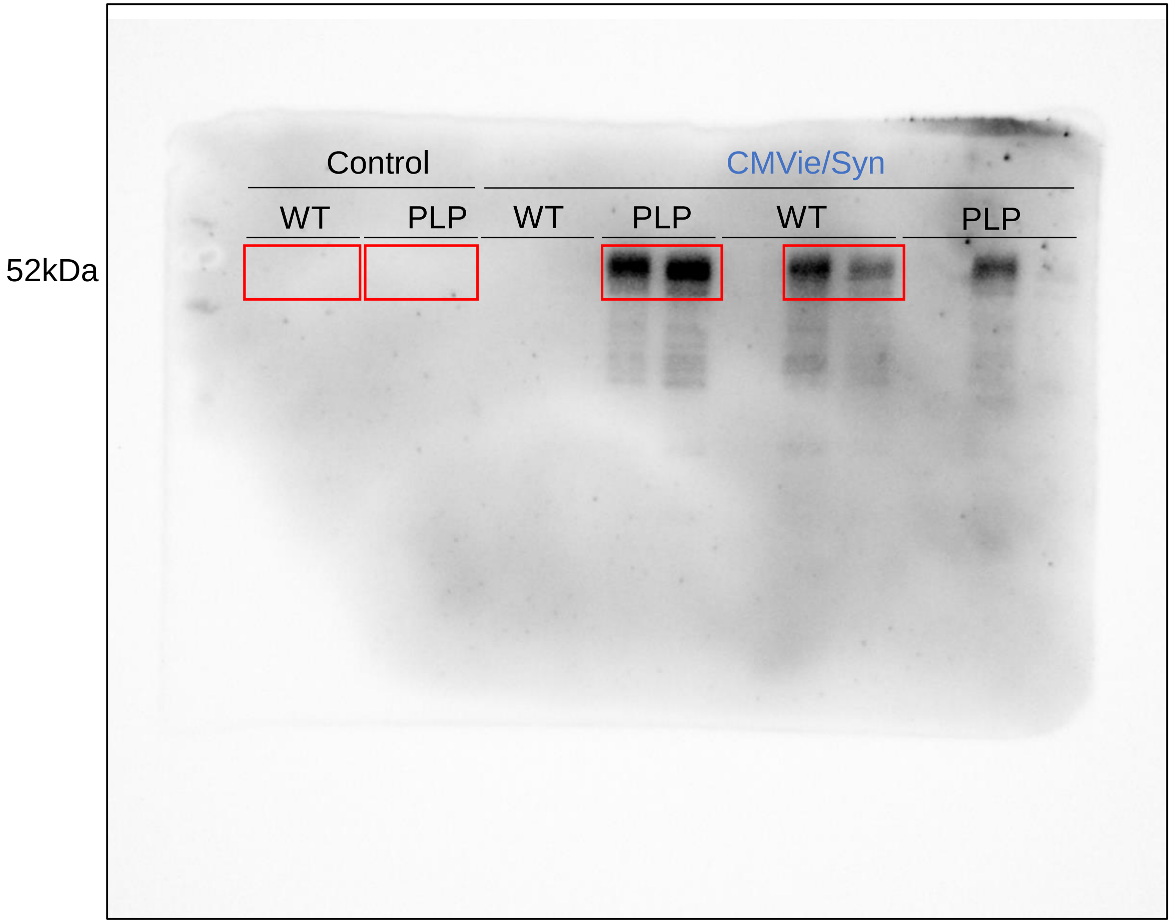

Actin

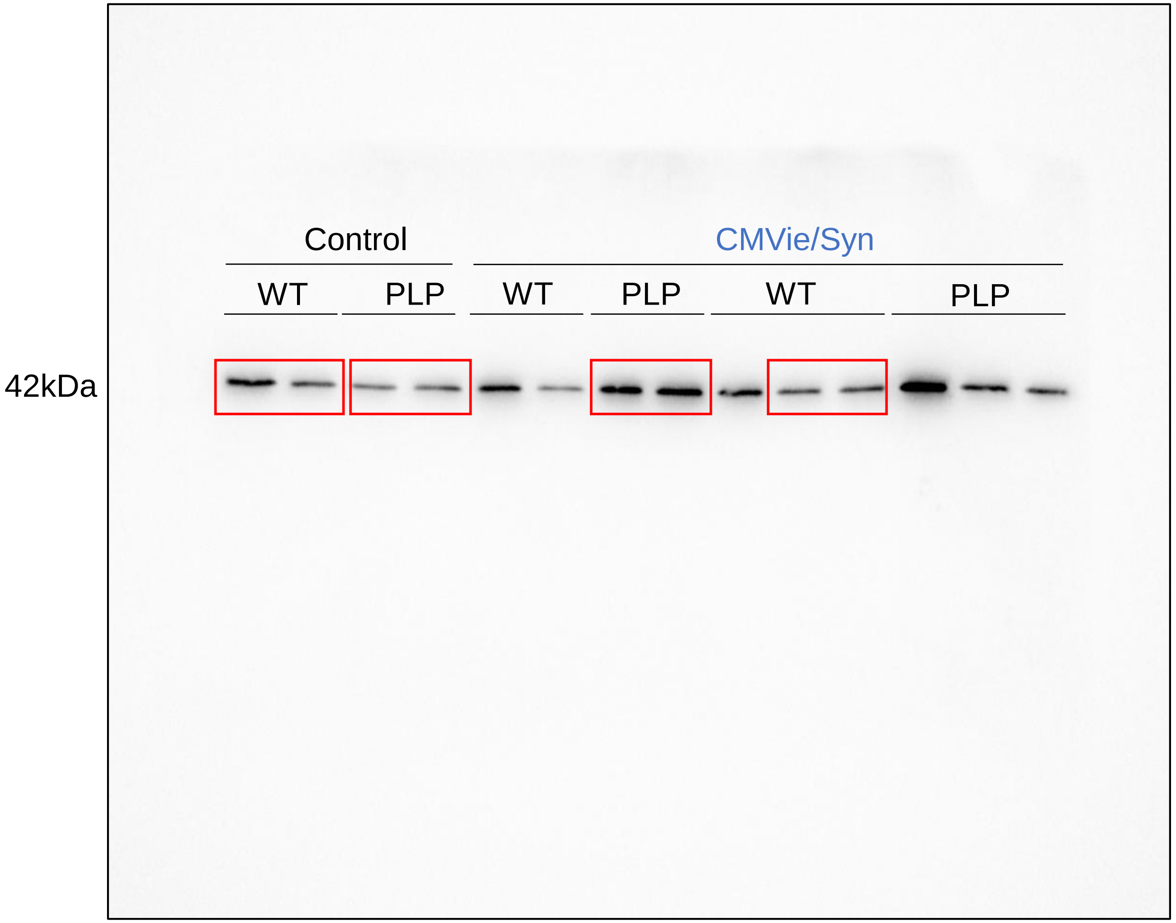

Full unedited gel for Figure 3F

Flag

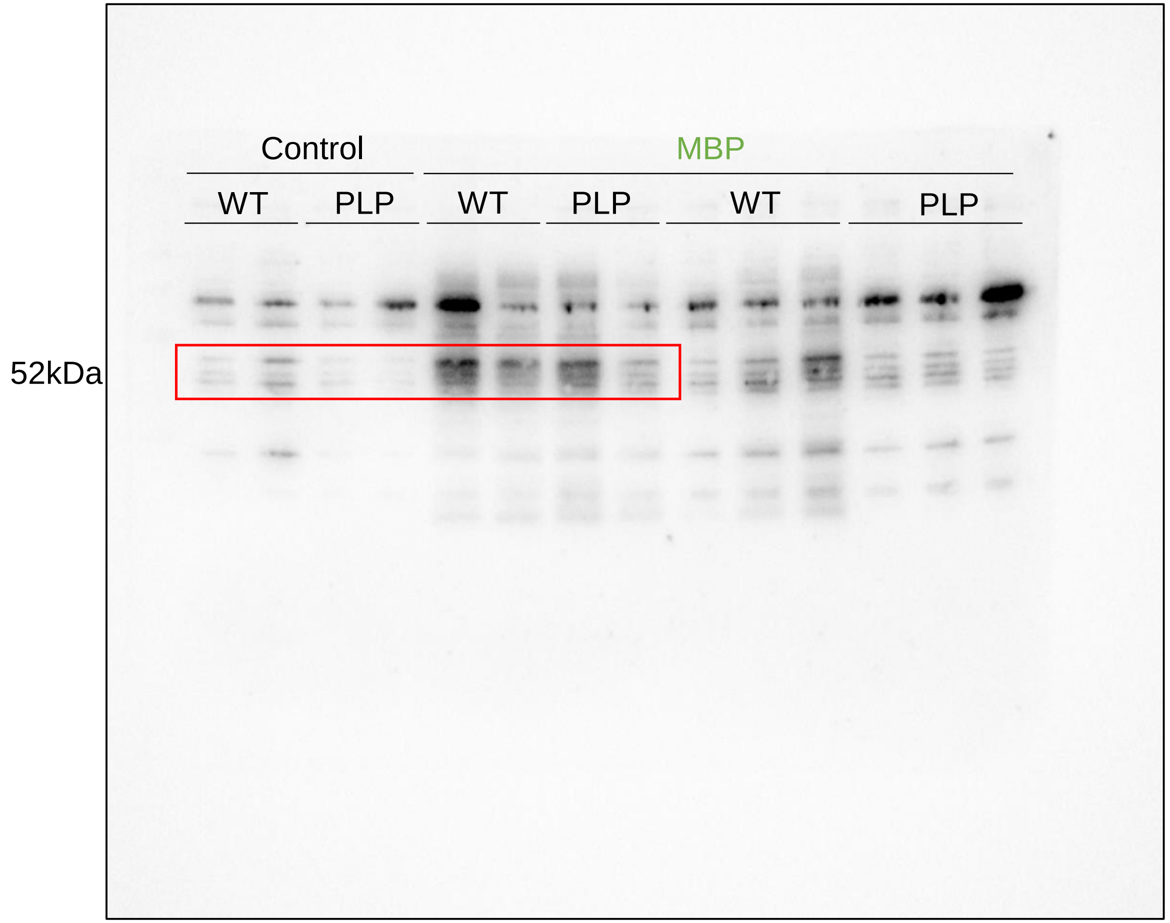

Actin

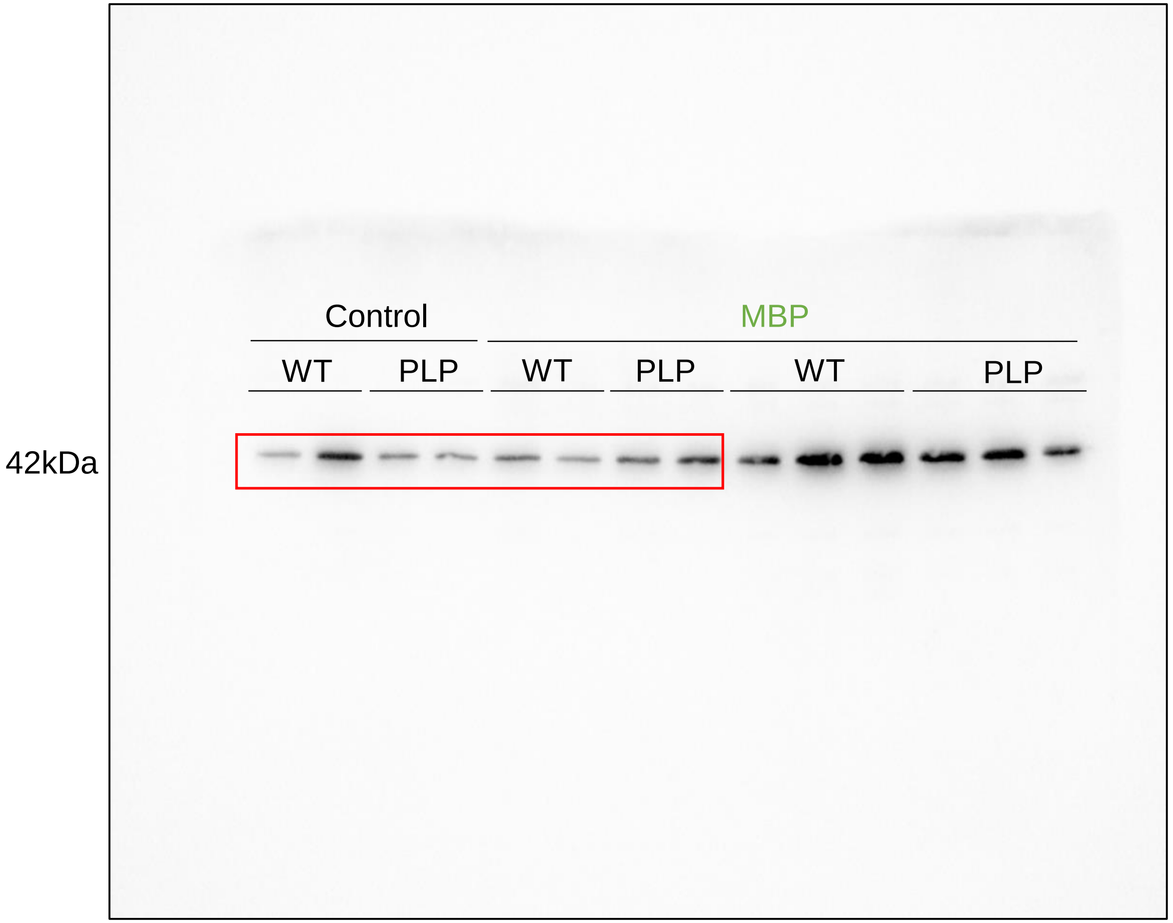

Full unedited gel for Figure 8B

CTSD

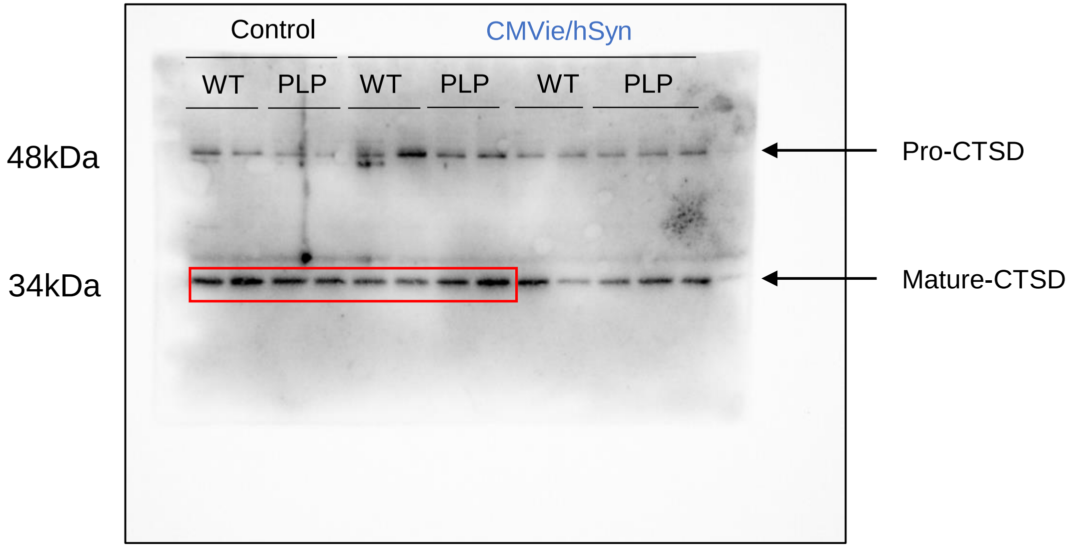

Actin

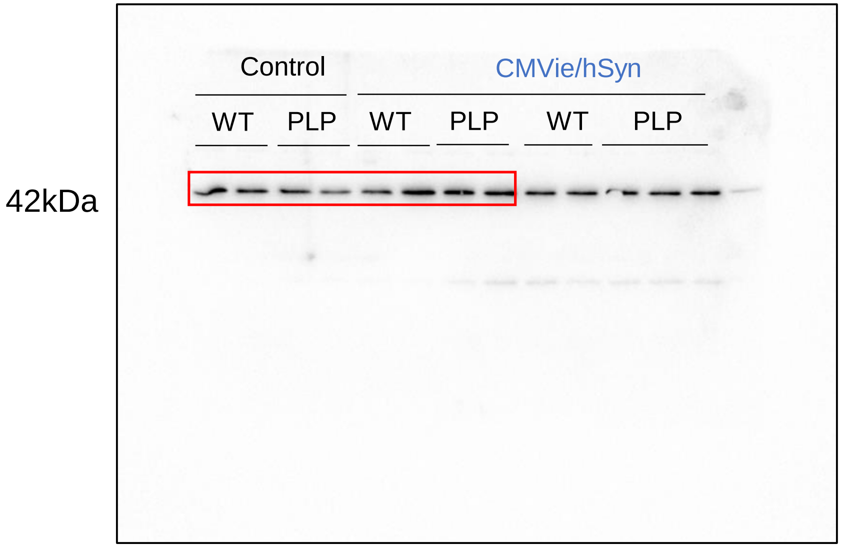

CTSD

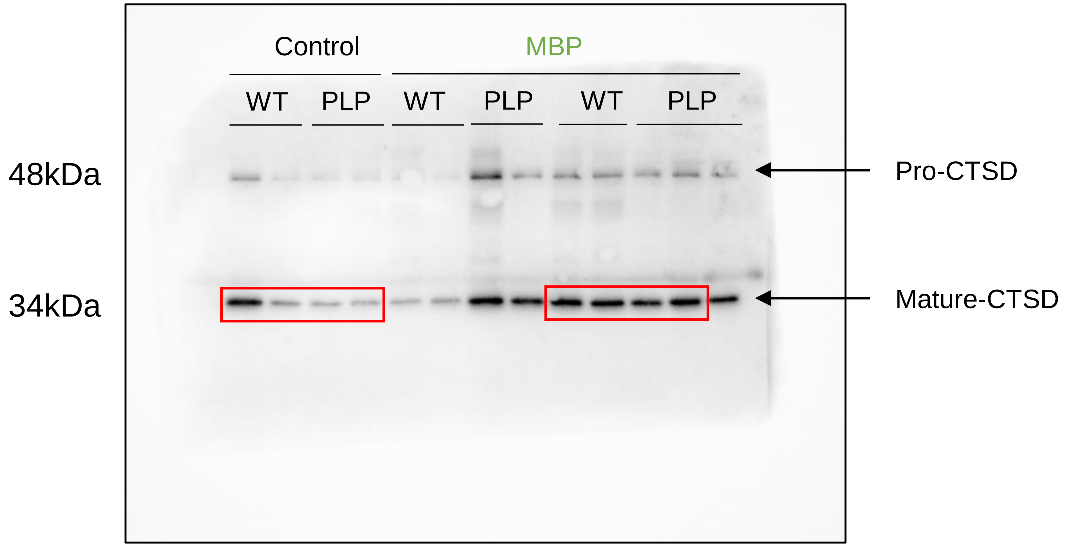

Actin

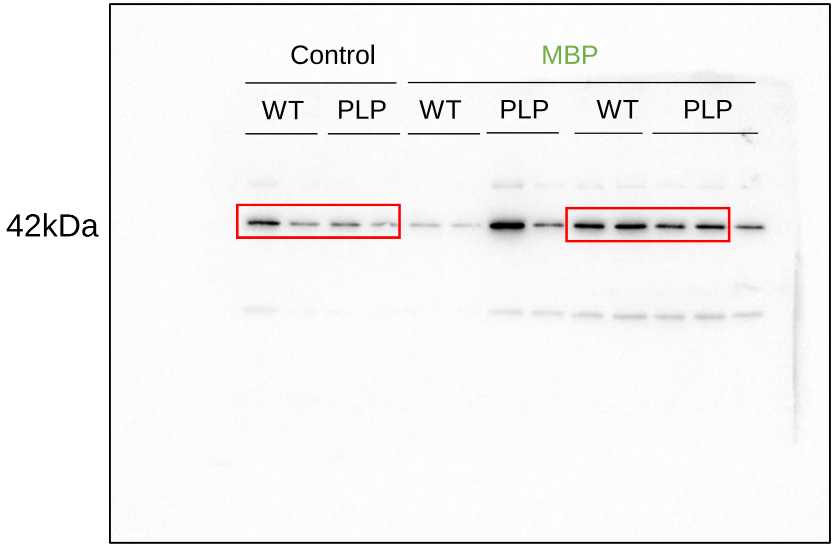

Full unedited gel for Supplemental Figure S3C

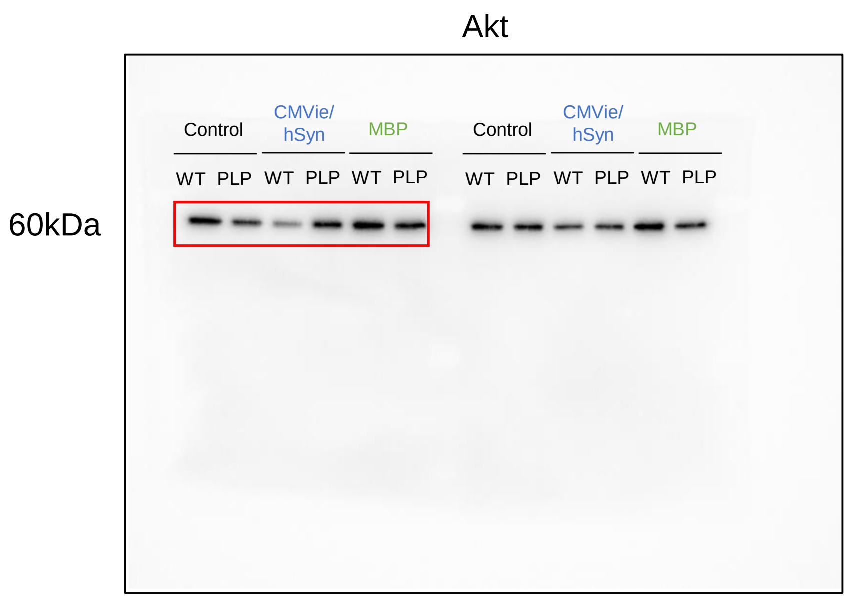

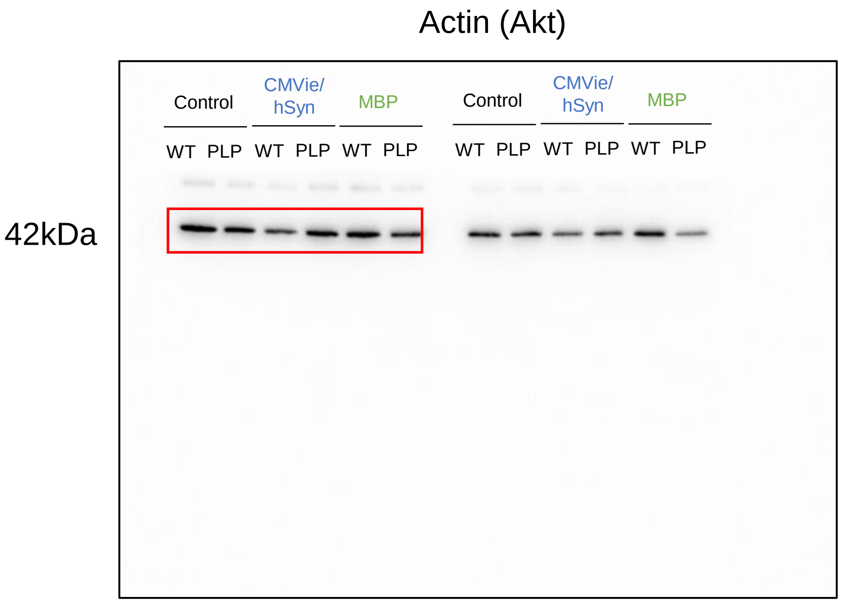

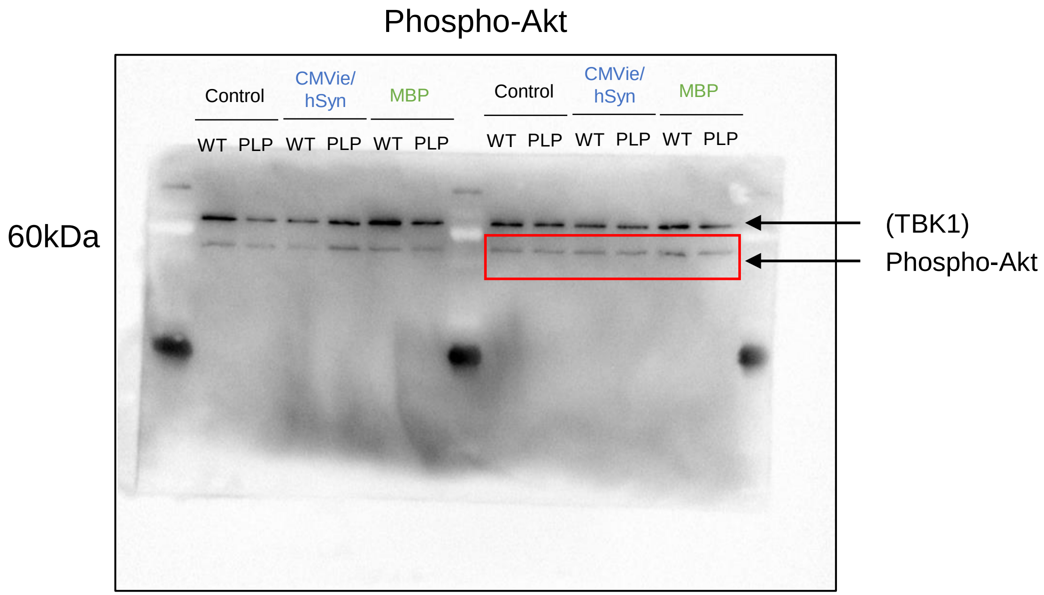

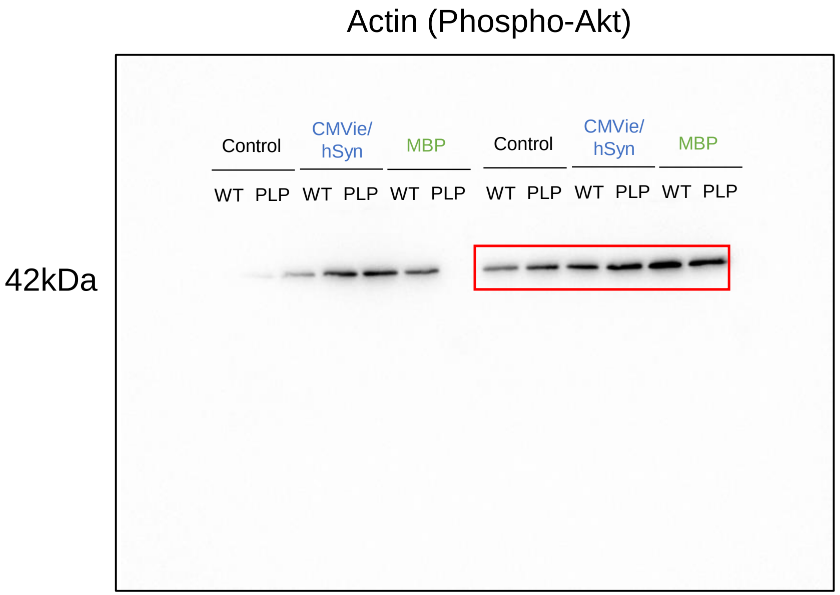

Supplement: Supplemental data [file jciinsight-4-129719-s109.pdf]
